# Human-modified habitats facilitate forest-dwelling populations of an invasive predator, *Vulpes vulpes*

**DOI:** 10.1038/s41598-017-12464-7

**Published:** 2017-09-25

**Authors:** Bronwyn A. Hradsky, Alan Robley, Ray Alexander, Euan G. Ritchie, Alan York, Julian Di Stefano

**Affiliations:** 1 University of Melbourne, School of Ecosystem and Forest Sciences, Creswick, VIC 3363 Australia; 2 Arthur Rylah Institute, Department of Environment, Land, Water and Planning, Heidelberg, VIC 3084 Australia; 3 Deakin University, School of Life and Environmental Sciences, Burwood, VIC 3125 Australia; 4 Present Address: School of BioSciences, University of Melbourne, Parkville, VIC 3010 Australia

## Abstract

Invasive and over-abundant predators pose a major threat to biodiversity and often benefit from human activities. Effective management requires understanding predator use of human-modified habitats (including resource subsidies and disturbed environments), and individual variation within populations. We investigated selection for human-modified habitats by invasive red foxes, *Vulpes vulpes*, within two predominantly forested Australian landscapes. We predicted that foxes would select for human-modified habitats in their range locations and fine-scale movements, but that selection would vary between individuals. We GPS-tracked 19 foxes for 17–166 days; ranges covered 33 to >2500 ha. Approximately half the foxes selected for human-modified habitats at the range scale, with some ‘commuting’ more than five kilometres to farmland or townships at night. Two foxes used burnt forest intensively after a prescribed fire. In their fine-scale nocturnal movements, most foxes selected for human-modified habitats such as reservoirs, forest edges and roads, but there was considerable individual variation. Native fauna in fragmented and disturbed habitats are likely to be exposed to high rates of fox predation, and anthropogenic food resources may subsidise fox populations within the forest interior. Coordinating fox control across land-tenures, targeting specific landscape features, and limiting fox access to anthropogenic resources will be important for biodiversity conservation.

## Introduction

Invasive and over-abundant predators are globally important drivers of ecological change^1,2^. Such predators can profoundly influence the structure of ecosystems, causing the decline and extinction of native prey, altering vegetation composition, and promoting other invasive species^1,3,4^. Subordinate, smaller-bodied predators (mesopredators) may become invasive or overabundant when removed from top-down control (mesopredator release); however, predator population dynamics can also be influenced by bottom-up processes^5,6^. In particular, human activities that alter habitat structure or improve resource availability can increase the abundance, survival, condition or hunting efficacy of generalist predators^6–8^. Indeed, anthropogenic disturbances and invasive predators may have synergistically negative impacts on native fauna^9,10^.

Understanding how invasive and over-abundant predators use human-modified habitats can therefore help improve predator management for biodiversity conservation. Knowledge of invasive predator habitat preferences, for example, may increase the efficacy of predator control: traps set along forest edges on the island of Guam caught three times more invasive brown tree snakes *Boiga irregularis* than traps set in forest interiors^11^. It can also inform alternative habitat-based predator management approaches. For instance, feral cats *Felis catus* in northern Australia select for grazed savannah over ungrazed savannah, and have four-fold greater predation success in open habitats; therefore managing livestock and fire to maintain vegetation cover could improve native prey persistence^12,13^. Similarly, controlling access to anthropogenic food resources can reduce over-abundant red fox *Vulpes vulpes* populations^14^.

Individual variation in predator use of human-modified habitats may also affect management success. Although much ecological research and theory characterises species as homogeneous units, generalist predator populations often comprise individual foraging specialists (e.g. feral cats^15^, badgers *Meles meles* ^16^ and coyotes *Canis latrans* ^17^). In such cases, using pooled or averaged values to describe traits such as resource selection can misrepresent populations^18^ and lead to inappropriate management decisions^19^. Likewise, models of community dynamics, including predator-prey interactions, can produce profoundly different outcomes if they incorporate individual variation^20,21^.

The red fox is a generalist predator whose range and abundance has increased substantially in modern times, due to intercontinental translocation, habitat modification and mesopredator release^22^. It is now invasive or over-abundant through much of its range, and is implicated in the decline and extinction of numerous smaller carnivore and prey species across Europe, North America and Australia^23–25^. Red foxes are highly adaptable and occur across a wide spectrum of natural and modified landscapes, from remote deserts^26^ to cities^27^. Nonetheless, red foxes readily exploit anthropogenic food sources^14^, and are more active and abundant in heterogeneous agricultural and developed landscapes than natural habitats^8,14,28^.

More than 90 studies have been published on red fox ranging behaviour^28^. Yet relatively little is known about how invasive red fox populations that dwell in predominantly natural landscapes (and therefore feed primarily on native fauna) use or benefit from human-modified habitats. Historically, control of red foxes for biodiversity conservation has had mixed success^29^, and better information on red fox habitat selection at multiple spatial scales could enable more efficient and scale-appropriate control^30,31^. Individual variation in habitat selection by invasive red foxes is not well understood, although differences in habitat selection preferences and activity patterns between individual foxes have been documented in Europe and Australia, albeit with small sample sizes^32–34^.

The aim of our study was to quantify broad- and fine-scale selection of human-modified habitats by invasive, forest-dwelling red foxes. Our two study regions (‘Otway’ and ‘Annya’) were situated in south-eastern Australia and predominantly comprised native forest; however, extensive road networks, water reservoirs, prescribed burns within the forests, and farmland and urban settlements along forest margins provided foxes with spatially heterogeneous access to human-modified habitats. We predicted that forest-dwelling foxes would (1) selectively locate their ranges to maximise access to human-modified habitats, and (2) select for human-modified habitats in their fine-scale movements, particularly at night (when most foraging occurs). We also expected that foxes would (3) show individual variation in their habitat selection preferences.

## Results

We obtained 18,217 GPS location data at 60-min intervals from 19 red foxes (9 female, 10 male; Table 1) after data screening. Data were collected over periods of 17–166 days (mean duration = 66 days), with 72–2437 successful fixes per individual (mean = 959; Table 1). We included data from all 19 individuals in the broad-scale habitat selection analysis, and quantified fine-scale selection for 15 individuals (6 female, 9 male) using 8013 movement step data.

**Table 1:** Red fox *Vulpes vulpes* individuals included in the analyses.

Fox ID	Sex	Mass	Est. age	Region	Session	Data collection	Successful 60-min fixes	99% BBMM (ha)		d_max_	Non-forest habitat
		(kg)	(months)			(duration in days)	(% successful)	day	night	(km)	(% availability)
LADY†^a^	F	4.1	>12 (lact.)	Otways	2	8 Nov – 1 Dec 13 (23)	280 (50%)	1118	1121	3.5	3%
VIKI†^a^	F	4.0	>12 (lact.)	Otways	2	26 Nov – 17 Dec 13 (21)	83 (16%)	1096	1296	2.7	4%
FERN†	F	2.9	6	Otways	1	29 Jan – 3 Mar 13 (2 reg. + 31 rotating)	32 (67%) incl. rotating 72 (51%)	7	43	1.0	4%
DUST	M	4.7	>12	Otways	2	26 Nov – 13 Dec 13 (17)	343 (84%)	457	265	2.3	7%
6010†^b^	M	4.5	>12	Annya	5	24 April – 3 Jun 15 (40)	555 (58%)	111	183	1.0	8%
4780^b^	F	3.5	>12	Annya	4	15 Jan – 5 May 15 (110)	2061 (78%)	236	353	1.5	10%
4190^b^	F	4.0	>12	Annya	4	15 Jan – 4 May 15 (108)	1848 (71%)	158	382	1.5	14%
GULY	M	3.7	6	Otways	1	22 Jan – 25 April 13 (3 reg. + 90 rotating)	65 (90%) incl. rotating 341 (86%)	12	230	3.2	18%
SAND	M	4.9	>12	Otways	2	23 Sept – 10 Nov 13 (48)	1076 (93%)	489	1473	5.0	18%
REED	M	5.3	>12	Otways	1	21 Feb – 7 Aug 13 (5 reg. + 161 rotating)	85 (71%) incl. rotating 357 (59%)	43	748	4.2	19%
CINN	F	4.5	10	Otways	3	17 May – 23 June 14 (37)	733 (82%)	89	141	0.8	21%
6410^b^	F	4.1	>12	Annya	4	15 Jan – 25 April 15 (100)	1943 (81%)	158	788	3.2	28%
RUSH	M	5.1	10	Otways	3	17 May – 15 June 14 (29)	638 (92%)	59	81	0.6	36%
RUST	M	4.9	?	Otways	3	17 May – 25 June 14 (39)	787 (84%)	34	303	3.5	38%
5590^b^	F	3.5	>12	Annya	5	26 April – 7 Jul 15 (72)	1340 (77%)	77	194	1.7	40%
4590^b^	M	4.0	>12	Annya	4	15 Jan – 14 May 15 (119)	2437 (86%)	154	298	1.8	45%
GAMY	M	5.4	≫12	Otways	3	17 May – 24 June 14 (38)	705 (77%)	234	94	1.6	49%
DOUG	M	4.4	>12	Otways	2	23 Sept – 3 Nov 13 (41)	914 (93%)	128	550	6.0	50%
4400^b^	F	5.0	>12	Annya	4	16 Jan – 15 May 15 (119)	2292 (80%)	1134	3040	6.7	72%

All individuals were included in the broad-scale habitat availability and selection analyses. The four foxes whose names are marked with † were not included in the fine-scale habitat selection analyses due to poor fix success rates. The *99* ***%*** *BBMM* is the 99% utilisation distribution from a Brownian bridge movement model; *d*_*max*_ is the maximum distance the fox travelled from the barycentre of its location data; *non-forest habitat availability* is the percentage cover of habitats other than native forest within a circle of radius d_max_ from the fox**’**s barycentre. ^a^These females were lactating and the antennae on their collars became badly damaged, presumably by cubs. This was probably the main cause of their low fix success rates. ^b^Mass for these individuals may have changed as they were caught and weighed considerably before the data collection period. Foxes 4190, 4400, 4590 and 4780 were caught in September 2014, and Foxes 5590, 6010 and 6410 in November 2014.

### Broad-scale habitat availability and selection

**Figure 1:**
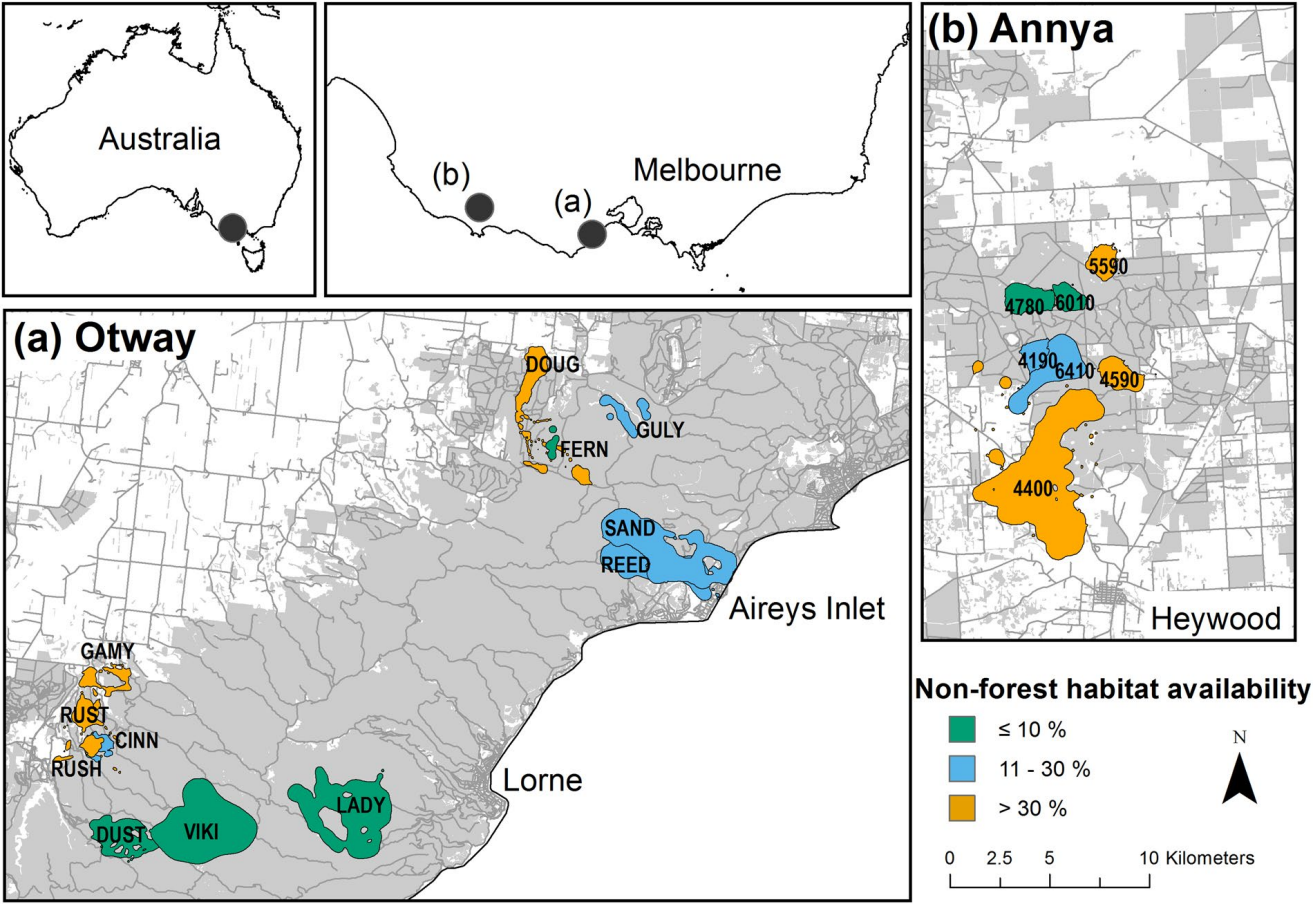
Locations of study regions and individual red fox *Vulpes vulpes* ranges, Victoria, Australia. Research was conducted in (**a**) Otway region Jan-2013 to Jun-2014, and in (**b**) Annya region Sep-2014 to Jun-2015. The 99% Brownian bridge movement model utilisation distribution (‘range’) of each individual is shown as a labelled polygon. Foxes in the same coloured cluster had similar access to non-forest habitats. Grey shading indicates tree cover, darker grey lines mark roads. Maps were generated in ArcMap 10.2^65^ using spatial layers listed in Supplementary Table S6.

All foxes were caught within native forest at Otway and Annya, Victoria, Australia. There was considerable variation in the relative availability of different habitat types among individuals, with foxes from both regions and sexes represented across the spectrum of habitat types (Fig. 1, Supplementary Table S1).

Six foxes had very little access to non-forest habitats (≤10% of available habitat). The 99% Brownian Bridge Movement Model utilisation distributions (hereafter ‘ranges’) of these foxes lay entirely within the forest boundaries (Fig. 1), and they travelled up to 3.5 km from their range centres (median d_max_ 1.9 km, range 1.0–3.5 km; Table 1). Three of these foxes did not use habitats selectively at this scale, two tended to use more young forest than random (one of these also avoided older forest), and one tended to select for reservoirs and against roads (Table 2).

**Table 2:**
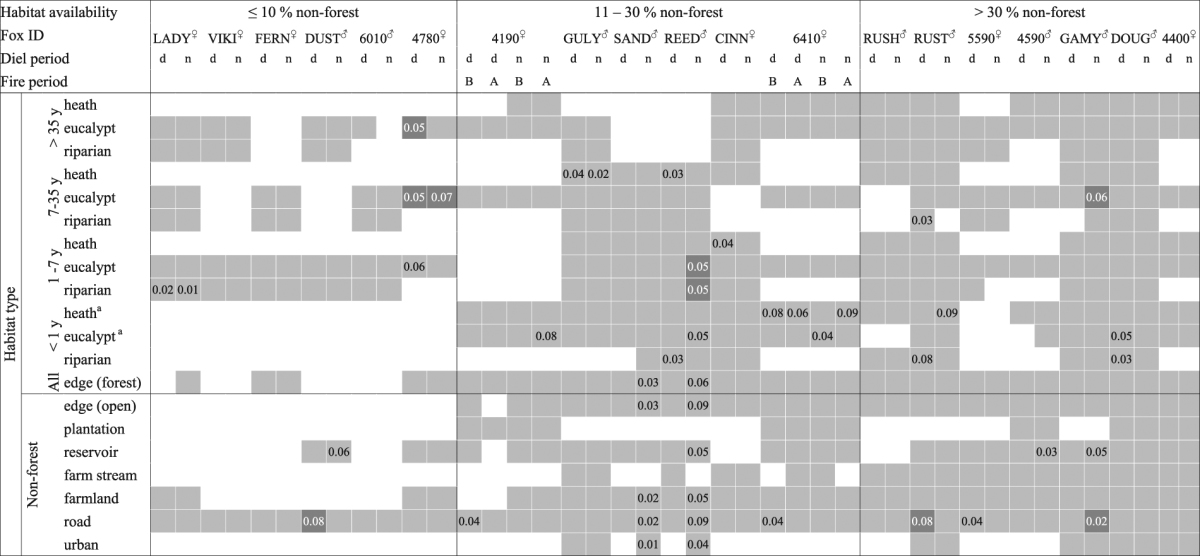
Habitat selection by red fox *Vulpes vulpes* in their diurnal (d) and nocturnal (n) range locations, Victoria, Australia. Numbers indicate the p-value for a one-way test of whether a habitat type was used more (light grey) or less (dark grey) than random. Habitats where both p-values were ≥0.1 are shaded light grey and no number is shown. Habitats that were not considered available to the individual were not tested and are not shaded. Selection data from before (B) and after (A) a prescribed fire were analysed separately for Foxes 6410 and 4190 to provide a temporal comparison. Superscripts indicate whether foxes were female (^♀^) or male (^♂^). Habitat use and availability data for all individuals are provided in Supplementary Table S2. ^a^For Foxes 4190 and 6410 before-fire (B) data, this refers to forest that would later be burnt but was >35 years old during pre-fire surveys.

**Figure 2:**
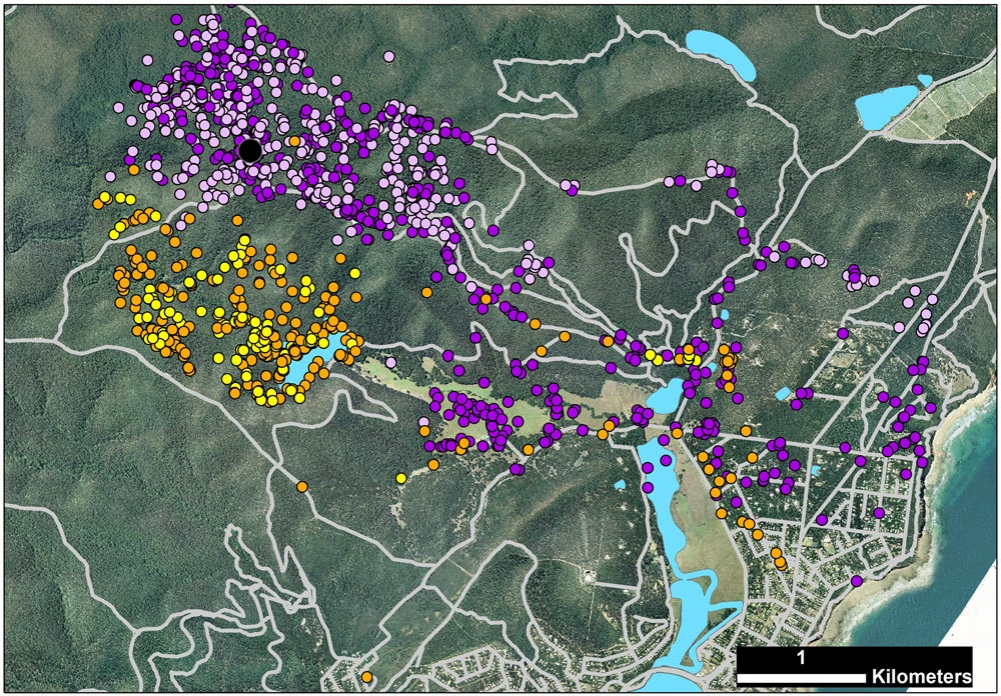
GPS location data for two adult male foxes *Vulpes vulpes* caught at the same location seven months apart, Otway Ranges, Australia. Fox REED (day - yellow; night - orange) was tracked Feb - Aug 2013, with data collected at 4–14 hour intervals and occasional bursts of 15 min intervals for 24-hours. Fox SAND (day – mauve; night - purple) was tracked Sept – Nov 2013 at regular 30 min intervals. Pale grey lines mark roads, blue shading indicates reservoirs and waterbodies, the large black dot marks the capture location. The map was generated in ArcMap 10.2^65^ using spatial layers listed in Supplementary Table S6. Aerial imagery courtesy Victorian State Government, Australia.

**Figure 3:**
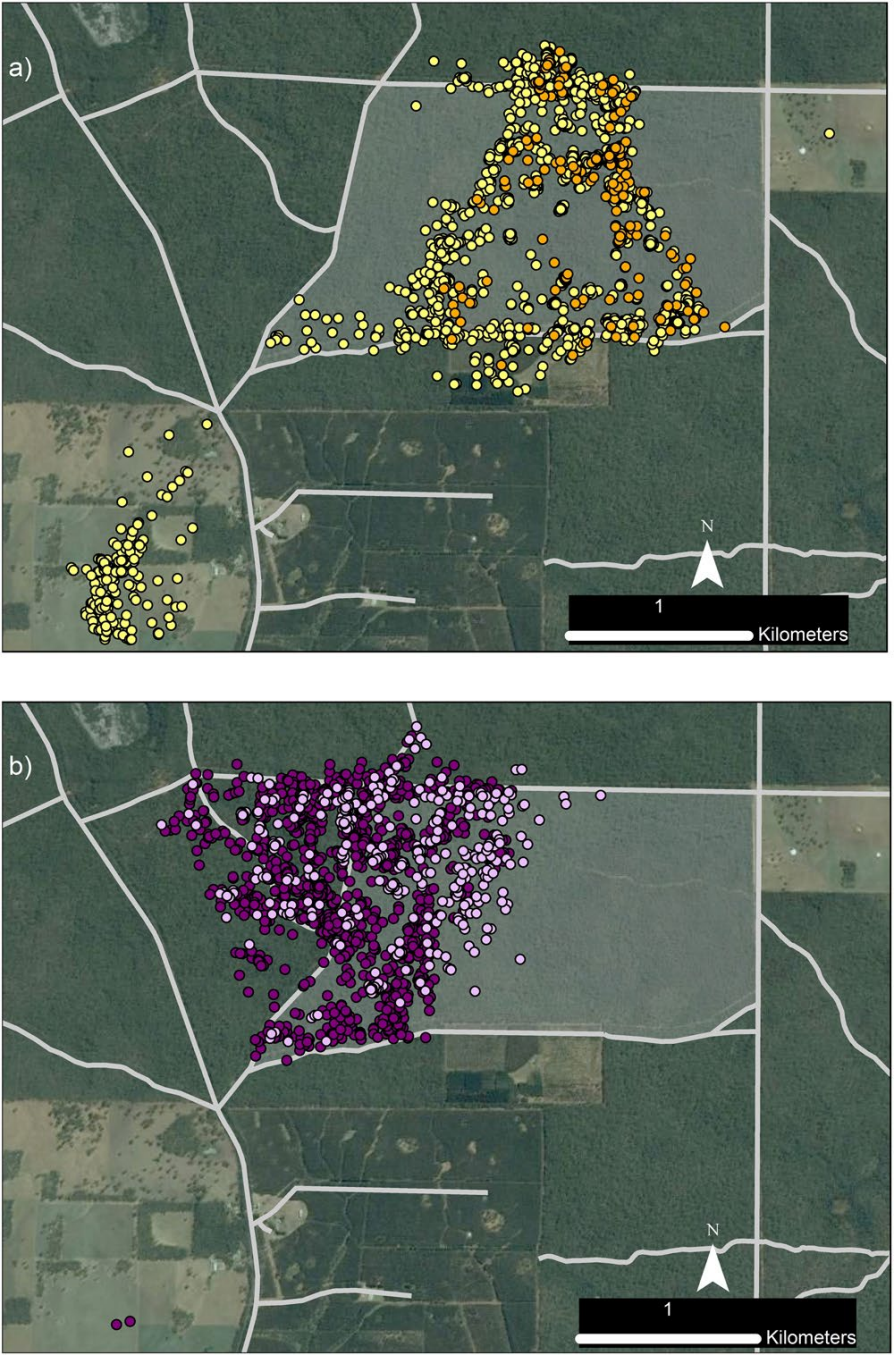
GPS location data of two adult female foxes *Vulpes vulpes* a) Fox 6410, b) Fox 4190, before and after a prescribed fire, Annya, Australia. In (**a**), yellow dots indicate pre-fire locations, orange dots indicate during and post-fire locations. In (**b**), dark purple dots indicate pre-fire locations, mauve dots indicate during and post-fire locations. Grey shading indicates the burn block; pale grey lines mark roads. Data were collected at 60 min intervals from 15-Jan-2015 and until 25-Apr or 4-May-2015, respectively. The prescribed fire was conducted 13–14 April 2015. Pre-fire, Fox 6410 consistently travelled to open farmland every two to three nights. Her last farmland visit was 9–10 April (three days before fire); she did not leave the forest again during the tracking period. Maps were generated in ArcMap 10.2^65^ using spatial layers listed in Supplementary Table S6. Aerial imagery courtesy Victorian State Government, Australia.

Another six foxes had moderate access to non-forest habitats (11–30% of available habitat). Several of these foxes had elongated ranges that extended beyond the forest boundaries or toward cleared areas within the forest (median d_max_ = 3.2 km, range = 0.8–5.0 km; Fig. 1, Table 1). Two individuals (SAND and REED) were mature males that were caught at the same location seven months apart. These males had similarly oriented territories (Fig. 2), and there was moderate to strong evidence that they selected for farmland, roads, forest-edges, open-edges and urban habitats in their nocturnal range locations; REED also tended to select for reservoirs, recently-burnt forest and mid-age heath, and against young eucalypt and riparian forest, while SAND did not shown any selection for these habitat types (Table 2). Two females with moderate access to non-forest habitats (foxes 6410 and 4190) selected for roads in their diurnal ranges. Following a prescribed fire, however, this selection ceased (Table 2). Instead, both females increased their use of burnt forest at night, with Fox 6410 nearly doubling the proportion of her nocturnal range that overlapped the burn block (33% to 64%; Fig. 3a), and Fox 4190 showing a smaller increase (37% to 45%; Fig. 3b). These females tended to select more strongly for burnt heath or eucalypt forest, respectively, after the fire (Table 2; Supplementary Table S2), and Fox 6410 stopped using farmland and edge habitats, which had previously comprised 25% of her nocturnal range. The decrease in selection for burnt eucalypt forest by Fox 6410 after the fire reflects an increase in availability (due to differences in range size) rather than a decrease in use (Table 2, Supplementary Table S2). The final two foxes with moderate access to non-forest habitats selected for young or mid-age heath at the range scale.

Seven foxes had ready access to non-forest habitats (>30% of available habitat), either because their ranges were close to the forest edge or because they travelled long distances from their range centre (median d_max_ = 1.8 km, range = 0.6–6.7 km; Fig. 1). Two of these foxes avoided roads, while another selected for them. Two also selected for reservoirs in their nocturnal ranges, and two tended to select for recently - burnt forest during at least one diel period. Mid-age forest was selected for by one fox but tended to be avoided by another. Two individuals with ready access to non-forest habitats did not show any selection at this scale.

### Fine-scale habitat selection

**Figure 4:**
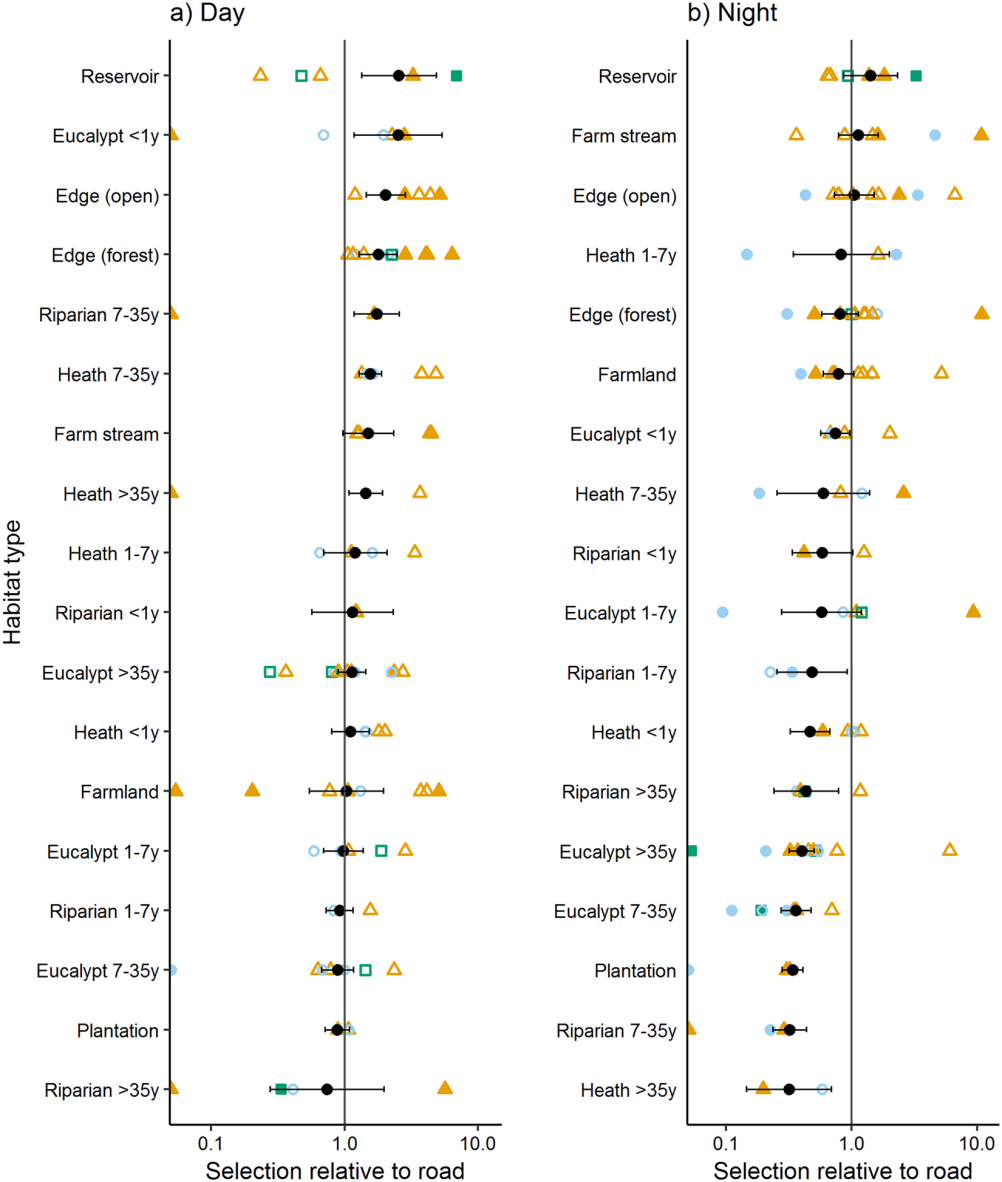
Odds ratios (ORs) from step-selection function models of red fox *Vulpes vulpes* movements during (**a**) day and (**b**) night. An OR > 1 indicates selection for the habitat type; an OR < 1 indicates selection against the habitat type, relative to the reference habitat type (road). The population-level analysis used 60-min interval GPS data from 15 foxes in Victoria, Australia, and is shown as a black circle with 95% confidence limits. Individual analyses were also conducted if more than 30 step data were available (n = 13), and are showed as filled points if the p-value of the OR was p < 0.05, or empty points if p ≥ 0.05. Colours indicate the availability of non-forest habitats at a broad scale: ≤10% = green squares, 11–30% = blue triangles, >30% = orange diamonds. Habitats that were available to an individual (i.e. comprised ≥ 2% random samples) but were never used are indicated by a point at 0.05. The habitat *urban* is not presented as it was not used by foxes during the day, and always comprised <2% of available samples in the individual analyses at night. Averaged across the population, the odds ratio for urban relative to road at night was 2.5 (0.56, 11.4).

Averaged across the population, the five habitats that foxes selected for most strongly in their fine-scale diurnal movements were water reservoirs, recently-burnt eucalypt forest, open-edges, forest-edges and mid-age riparian forest. At night, the most highly selected habitats were urban settlements, water reservoirs, farm streams, open-edges and roads (Fig. 4; Supplementary Table S3). The largest decreases in selection between day and night occurred for mid-age riparian forest and long-unburnt heath, while the greatest increases were for roads and farmland (Fig. 4; Supplementary Table S3).

There was some evidence that two female foxes tracked before and after a prescribed fire selected for forest within the burn-block more strongly post-fire; however, small post-burn sample sizes meant that confidence intervals around these estimates were large. For example, the odds ratio of selection for heath in the burn block relative to road for Fox 6410 at night was 0.5 (0.3, 0.9) pre-fire and 1.0 (0.3, 3.2) post-fire (Supplementary Table S4). In the Otway region, four foxes caught in or near a burn block approximately six weeks after an intense prescribed fire, used but did not strongly select for recently-burnt heath or eucalypt forest in their fine-scale movements (Fig. 4, Supplementary Table S4).

Within the overall population patterns, there was substantial variation in fine-scale habitat selection among individuals (Fig. 4, Supplementary Table S4). For example, at the population-level, there was little difference between selection for roads and open-edges at night (Fig. 4); however, fox SAND strongly selected for roads over open-edges (mean odds ratio [LCL, UCL] = 2.3 [1.4, 4]), and fox CINN strongly selected for open-edges over roads (3.4 [1.8, 6.3]). Likewise, water reservoirs were strongly selected for by most foxes that had access to them, but reservoirs were among the lowest ranked habitats of Fox 4400, despite similar availability (Supplementary Table S4).

## Discussion

The red fox is a quintessential invasive mesopredator. Highly adaptable, it has benefited from human activities that have provided release from both top-down and bottom-up trophic control^14,22,35^. Human-modified habitats appeared to facilitate invasive red fox populations across the predominately natural forest landscapes we studied. Many individuals selected for fragmented and disturbed habitats such as forested and open edges, recently-burnt forest and water reservoirs at broad and/or fine spatial scales, indicating that native fauna in these habitats are likely to experience high rates of predation. Furthermore, anthropogenic resources may be subsidising fox populations well within the forest interior, as some foxes travelled long distances from the core of their forest ranges to the forest-farmland interface, expedited by movement along roads. A sound understanding of the behavioural ecology of invasive species (including intraspecific variation) is needed to improve pest management for biodiversity conservation.

Invasive and over-abundant generalist predators often thrive in fragmented or otherwise human-modified habitats^1,7^. Foxes in our landscapes exhibited a variety of ranging behaviours: some individuals were confined to the native forest, others had elongated ranges that covered both forest and cleared land, while others had compact ranges on the forest edge. Our prediction that invasive forest-dwelling foxes would selectively locate their ranges to maximise their access to human-modified habitats was partially supported. Four of the nineteen foxes did not show any selection at the range scale, but most others selected for roads, reservoirs, forest edges and/or recently-burnt or young forest in their range locations. Selection for older forest by several individuals, particularly during the day, contradicted our prediction, but was consistent with the species’ use of complex habitats for diurnal shelter^36^. The general orientation of ranges toward human-modified habitats suggests that these habitats may be providing important resources for invasive forest-dwelling foxes. Elsewhere, foxes are known to regularly travel up to 8 or even 12 km to access highly-concentrated sources of anthropogenic food such as feeding stations^37^, ski resorts^38^ or vineyards^39^. However, foxes whose home range centres are more distant from these resources do not make foraging trips^37^. Thus, human-modified habitats outside the forest may be providing important resources for invasive forest-dwelling foxes in our study landscapes, but only within a restricted distance of the forest edge.

Our second prediction that forest-dwelling foxes would select for human-modified habitats in their fine scale movements, particularly at night, received more consistent support. Averaged across the population, foxes selected strongly for human-modified habitats such as water reservoirs and forested and open edges during both diel periods, and intensified their selection for highly-modified habitats such as roads and farmland at night. Generalist predators may be attracted to forest edges by high prey densities^40^ and/or increases in prey vulnerability associated with fragmented and open habitats^12,41^. In our landscape, water reservoirs, forest edges, riparian strips in farmland, and roads were likely to provide rich foraging resources for foxes, including native waterbirds, small mammals, introduced European rabbits (*Oryctolagus cuniculus*), grazing macropods and road-killed animals (B. Hradsky, pers. obs.). Some of these habitats also provided human refuse: several carcass dumps and rubbish skips were located along forest edges and were regularly visited by foxes (B. Hradsky, unpublished data). Foxes that ranged through open farmland repeatedly visited small areas over several days, perhaps indicating the presence of an animal carcass (B. Hradsky, unpublished data). More information on native and introduced prey occurrence in these habitats, together with higher resolution mapping of anthropogenic resources, would improve our understanding of fox behaviour, habitat selection and the role of resource subsides in forest fox ecology.

Selection for roads at both spatial scales suggests that roads were an important resource for some foxes. Positive associations between roads and red fox occurrence have been demonstrated elsewhere in Australia, Europe and North America^34,42–44^, and May and Norton^45^ suggested that roads might enable invasive predators to access otherwise remote native prey populations. We propose, however, that roads in our study regions provided foxes with access in the other direction: most foxes spent their days within the forest and increased selection for roads and other non-forest habitats at night. Foxes often travelled rapidly along roads for more than an hour and repeated the same route on different occasions (B. Hradsky, unpublished data). By enabling foxes to travel long distances to human-modified habitats, roads may be extending the radius of anthropogenic resource subsidisation further into the forest.

Our data on red fox responses to fire are limited, but show that two foxes intensified their use of burnt forest immediately after a prescribed burn, while several others selectively used forest that had been burnt 1.5–12 months previously. Similarly, a fire experiment in the Otways found that invasive predator occurrence increased in burnt forest in the weeks following fire^46^. Predators may be attracted by, or intensify their use of, recently burnt habitats if prey abundance or vulnerability to predation increases post-fire^13,47^, and will sometimes make long extra-territorial movements to fire scars^48^. In our study, however, responses appeared highly localised: after the prescribed fire at Annya, only the two foxes whose pre-fire home ranges overlapped the burn-block used the burnt forest; five other individuals that lived within four kilometres of the burn edge showed no response. Similarly localised responses to grassland fire have been observed in swift fox *Vulpes velox* ^49^. Given that high levels of post-fire predation may affect native fauna recovery in burnt landscapes^50^, additional longer-term, before-after GPS tracking experiments are required to better understand the factors affecting invasive predator responses to fire.

Selection for human-modified habitats by forest-dwelling foxes is likely to impact native fauna both within these habitats (where many threatened species persist^51^), and across the broader forest landscape. Intense use of disturbed, fragmented and remnant habitats by invasive red foxes may expose native animals in these environments to greater predation risk. This may explain why prey species are more vulnerable to red foxes in landscapes that contain a mix of forest and cleared land than in homogenous forest or farm landscapes^41,52^. Moreover, it could increase the risk of habitats such as vegetation corridors and recently-burnt forest becoming population sinks for native fauna. Interactions between threatening processes such as habitat fragmentation and predation by invasive species can compound the risks to native fauna, and may require coordinated management^9,10^ Access to anthropogenic resource subsides such as human refuse and domestic animals could also increase invasive predator abundance and survival^7^, leading to ‘spillover’ predation of native fauna in the surrounding area^53^. Furthermore, fox populations may be released from bottom-up control if they are able to access supplementary sources of food, allowing them to continue depredating native fauna even when prey populations are very low, i.e., hyperpredation^54^.

Individual variation and specialisation are being documented in an increasing number of species, particularly upper trophic-level predators^55^, and have important implications for community ecology^21^. Consistent with our third prediction, we found that although fox habitat selection tended to follow some general patterns, there was substantial variation between individuals. Individual variation and behavioural plasticity in species complicates management, as any protocol is unlikely to be universally applicable^56^. For instance, targeted baiting of habitats such as roadsides and farm streams might increase bait-uptake by foxes^31^, but would not control the entire fox population, as some individuals avoid these habitat types. Similarly, rabbit control is unlikely to protect endangered birds from hyperpredation by feral cats because not all cats select for areas with abundant rabbits^19^. More broadly, further research is needed to elucidate how habitat selection by predators varies with population demographics and environmental context.

In conclusion, most forest-dwelling foxes in our study regions had access to and selected for human-modified habitats at broad or fine spatial scales. Nonetheless, scat analyses show that foxes in these regions feed heavily on native fauna, particularly mammals^46,57^. If resources from human-modified habitats are helping sustain abundant invasive predator populations, predator control for biodiversity conservation will need to be conducted at a broad landscape scale and coordinated across land tenures. Targeting specific landscapes features such as forest-farmland edges, water reservoirs, roads and recently-burnt forest may increase the efficacy of fox control programs, but individual variation in habitat preferences must also be considered. Finally, habitat-based approaches that limit fox access to anthropogenic resources (such rubbish skips and animal carcasses) may provide an alternative or complementary way of reducing red fox populations and protecting native fauna in predator-invaded landscapes.

## Methods

Experimental protocols for Otway and Annya were approved by the University of Melbourne Animal Ethics Committee, and the Department of Environment, Land, Water and Planning Animal Ethics Committee, respectively. All research was conducted in accordance with the applicable institutional, state and national guidelines and regulations for the care and use of animals. Details of animal ethics approvals, research permits and pest animal permits are provided in Supplementary Table S5.

### Study area

Our study was conducted across two forested regions in south-eastern Australia: Otway (38°24′ S, 144°1′ E) and Annya (38°07′S, 141°15′E) – Fig. 1. The climate in both regions is temperate, with cool wet winters and dry warm summers. There is an elevation, rainfall and vegetation gradient across the Otway region: the north-east is relatively flat and dry (150–250 m a.s.l, 800–1000 mm average annual rainfall), and dominated by heathy woodland and lowland eucalypt forest, whereas the south-west is 400–550 m a.s.l., receives 1300–1600 mm average annual rainfall and is dominated by shrubby wet eucalypt forest and cool temperate rainforest^58,59^. The Annya region is 70–150 m a.s.l. and has a mean annual rainfall of 835 mm; the vegetation is similar to that of the north-eastern Otways. Red foxes are the largest mammalian predator in both regions, and feed heavily on native fauna^46,57^. Forested areas in both regions are protected as national and state park.

Large wildfires affected Otway and/or Annya in 1939, 1968 and 1983, and smaller prescribed burns have been conducted throughout the forests, particularly in the last seven years. Thus there are four broad forest age categories: recently-burnt (<1 year post-fire), young (1–7 years post-fire), mid-age (7–35 years) and long-unburnt (>35 years post-fire).

### Fox capture and handling

We conducted the study over five trapping sessions between Jan-2013 and Nov-2014, with tracking continuing until Jun-2015. We captured foxes using padded offset-jaw foothold traps (no. 1.5) set within the forest, and baited with a variety of food, scent and visual lures. Food lures were buried to avoid attracting non-target animals. Trapped foxes were restrained with a catch-pole and sedated via an intramuscular injection of anaesthetic/analgesic. They were then checked for injury, sexed, weighed, and the foot that had been trapped was massaged with cinchocaine hydrochloride 5 mg/g, zinc oxide 200 mg/g (Rectinol). Healthy individuals that met the body mass criteria were fitted with a GPS collar with drop-off mechanism (collars weighed < 5% of body mass in all cases). A reversal agent for the sedative was administered intramuscularly, and the individual placed in a sheltered location at the site of capture to recover. Each fox was given a unique four-digit identification code, either alphabetic (Otway) or numeric (Annya). Details of sedatives, collars and reversal agents are provided in Supplementary Table S5.

### Data collection

Data collection schedules varied between trapping sessions. In session one (Otway), collars had a variable rotating schedule, with 36 fixes collected at 4–14 hour intervals over 12 days, interspersed by 24 hour periods during which data was collected at 15 min intervals. In sessions two and three (Otway), fixes were collected at 30 min intervals; in sessions four and five (Annya), fixes were collected at 60 min intervals. Fix schedule and data retrieval details are provided in Supplementary Table S5.

### Data analysis

#### Data cleaning and pre-processing

Location data were discarded if collected while trapping was still being conducted, when the collar battery was severely depleted, or on the day of collar drop-off. Regular interval data were screened^60^ to remove fixes that reflected a turning angle of 170–190° and travelling speeds >500 m/h (indicating an erroneous ‘spike’), or that exceeded mean and median distance criteria (see Supplementary Table S5). Rotating schedule data from session one were visually checked to remove obviously erroneous fixes. All data except the rotating schedule data from Session One were then thinned to 60 min intervals to provide consistency during data analysis.

#### Defining habitat features

Based on previous literature on red fox ecology^31,42,61,62^, we identified human-modified habitats and other landscape features that (a) we predicted would influence fox habitat selection and (b) could be derived from geographic information system map layers. These included forest age, forest type, edges between forests and open farmland (differentiated as the forest- or open-side of the edge), water reservoirs and dams, streams, roads, farmland, and urban settlements. We classified the study area accordingly; descriptions and derivations of the 20 habitat types are provided in Supplementary Table S6. For most foxes, habitats did not change during the tracking period. However, a prescribed fire was conducted within the ranges of two individuals at Annya during the tracking period. We analysed before- and after-fire habitat selection separately for these individuals so that pre-fire selection of forest within the burn block could be used as a temporal control. It was not feasible to include a habitat × time interaction effect because some habitats were never used post-fire.

#### Habitat selection and availability analysis

Our study regions were spatially heterogeneous and large relative to the foxes’ movement capabilities, and so we expected that different individuals would have access to substantially different habitat bases. Habitat availability can affect selection (i.e. functional responses^63^), and we also anticipated that red fox habitat preferences would differ between day and night^64^. Mixed models with random intercepts and random coefficients can be used to control for unbalanced designs and functional responses^18^, but it becomes difficult to account for functional responses to several habitat types simultaneously. As mentioned above, the inclusion of interaction terms (such as the effect of diel period on habitat selection) also requires a habitat to be available and used at least once in each period. We therefore chose to run separate analyses for each individual’s habitat selection preferences during each diel period so that individual and diel effects on habitat selection could be clearly identified. We also ran population-level models of fine-scale diurnal and nocturnal selection. We distinguished day and night location data using date-appropriate sunrise and sunset times.

*Broad-scale habitat availability:* To quantify the habitats that were broadly available to foxes, we defined a circular polygon for each fox that was centred on the barycentre (mean easting and northing) of its location data (using the 60-min interval data for all individuals plus the rotating schedule data for individuals caught in session one) and had a radius of the maximum distance the fox had travelled from this point (d_max_). These values were calculated from pre-dispersal data for two individuals (CINN and RUST) who dispersed during the tracking period (as indicated by a sudden spike in range area, when plotted against sequential fix collection date). For each fox, we then calculated the proportion of the circle that comprised each habitat type. The small sample size (19) relative to the number of habitat variables (20) precluded a formal cluster analysis of habitat availability. For ease of interpretation in figures, we grouped foxes into three classes, based on their access to habitats other than native forest using natural breaks (Jenks) classification in ArcMap 10.2.1^65^.

*Broad-scale habitat selection:* To examine whether foxes selectively located their ranges within the landscape, we defined broad diurnal and nocturnal utilisation distributions (hereafter, ‘ranges’) for each fox using 99% Brownian bridge movement models (BBMMs^66^). The large percentile (99%) ensured that the ranges were relatively contiguous and captured most movement pathways between consecutive fixes. BBMM calculations were restricted to fixes collected at 60-min intervals, and used an estimated location error of 20 m and a grid-cell size of 20 m. Again, dispersal data were discarded for two individuals. There was no evidence that the overall range area was correlated with either the number of fixes (*r*_*s*_ = 0.24, *p* = 0.33) or duration of data collection (*r*_*s*_ = 0.34, p = 0.16).

We then compared the composition of habitats within each fox’s nocturnal and diurnal range to the composition of habitats within 100 randomly located range samples (to represent habitat availability). To create the random samples for each individual, we randomly relocated the trajectory of its location data 100 times. This involved rotating the trajectory at a random angle around the barycentre, and then transposing the barycentre of the new trajectory by a random distance between 0 and d_max_ from the original barycentre. Transpositions were constrained so that trajectories never overlapped the ocean. We then defined the 99% BBMM nocturnal and diurnal ranges for the transposed trajectory, and calculated the area that comprised each habitat type. For each fox, we tested whether the proportion of its range that comprised each habitat type was greater or less than that available in the randomly sampled ranges. Tests were conducted using separate one-way tests as data violated the two-way test assumption of a symmetric random distribution. Results are only presented for habitats where the 97.5% quantile of the random samples was ≥ 2%.

*Fine-scale habitat selection:* To examine fine-scale habitat selection by foxes as they moved through the landscape, we used a step selection function with conditional logistic regression^67,68^. This approach compares the habitat at the destination of each observed moving step (used) to the habitat at the destinations of *n* matched control steps (available); a strata variable is used to pair the actual step with its matched controls. The habitats that are considered available to an individual are therefore varied as a function of its current location, rather than assuming that the entire home range is available within a single time step^69^.

To prevent any confounding effect of fix interval, we only used 60-min interval data for this analysis. We also discarded fixes that were ≤ 28 m from the previous fix, as activity sensor data from the collars indicated that foxes were moving < 50% of the time when these fixes were collected. Finally, we excluded data from four foxes that had fix success rates less than 70% (Table 1) to reduce any potential biasing effect of habitat type on fix success.

For each observed step, we generated 20 control steps in Geospatial Modelling Environment^70^ by randomly sampling step lengths and turning angles from the observed data. We then used ArcMap 10.2.1^65^ to identify the habitat type at the destination of each observed and control step. Prior to analysis, we grouped habitat types that occurred at less than 2% of an individual’s control step destinations with similar, better-represented habitat types.

To derive population-averaged estimates of habitat selection for each diel period, we used a generalised estimating equation with robust standard errors based on data from all fifteen individuals with fix success rates >70%. We also constructed separate diurnal and nocturnal models for individuals if more than 30 observations were available. For the individual analyses, habitat types that were never used by the individual during the diel period but comprised at least 2% of control step destinations were dropped from the analysis so that the model would run, but are indicated in the results. The results for the population- and individual-level analyses are presented using road as the reference level, as road was the only habitat type that was used by all individuals in both diel periods. Odds ratios and confidence intervals for all habitat contrasts are presented in Supplementary Tables S3 and S4.

We have not adjusted for comparison-wise error rates, but instead present confidence intervals and effect sizes as well as p-values, allowing the reader to make their own judgements about the ‘significance’ of our findings. When full estimates and confidence intervals were too bulky to include in the main text, they are provided in the supplementary material.

#### Software

Unless otherwise specified, analyses were conducted in R^71^. R packages and citations are detailed in Supplementary Table S7.

### Data availability

The GPS datasets generated and analysed during the current study have been uploaded to Movebank (www.movebank.org; Otways ID 313263057; Annya ID 313789633).

## Electronic supplementary material

Supplementary Information Tables S1, S4 - S7

Supplementary Information Table S2

Supplementary Information Table S3
