# Supplementary material for: Human-modified habitats facilitate forest-dwelling populations of an invasive predator, Vulpes vulpes
Source: Sci Rep. 2017 Sep 25;7:12291. doi: 10.1038/s41598-017-12464-7 (PMC5612948; doi:10.1038/s41598-017-12464-7)
Supplement: Supplementary file 1 — Supplementary Information Tables S1, S4 - S7 [file 41598_2017_12464_MOESM1_ESM.pdf]

SUPPLEMENTARY INFORMATION

Human-modified habitats facilitate forest-dwelling populations of an invasive predator, *Vulpes vulpes*

Hradsky BA*, Robley A, Alexander R, Ritchie EG, York A, Di Stefano J.

* hradskyb@unimelb.edu.au

Supplementary Table S1. Availability of habitat types to individual red fox *Vulpes vulpes*, Victoria, Australia. Numbers show the percentage of each habitat type within a circle defined by the fox's barycentre and maximum ranging distance (see main text for details).

		LADY♀	VIKI♀	FERN♀	DUST♂	6010♂	4780♀	4190♀	GULY♂	SAND♂	REED♂	CINN♀	6410♀	RUSH♂	RUST♂	5590♀	4590♂	GAMY♂	DOUG♂	4400♀
Long-unburnt forest (>35 y post-fire)	heath	0	0	0	0	0	0	0	0	0	0	0	2	1	6	0	5	1	0	1
	eucalypt	35	25	0	60	1	30	35	10	0	0	6	32	2	11	3	38	5	6	15
	riparian	29	48	0	22	0	0	0	2	0	0	3	0	1	4	1	0	1	1	0
Mid-age forest (7-35 y post-fire)	heath	0	0	0	0	0	0	0	19	6	6	4	0	8	2	0	0	0	5	0
	eucalypt	3	0	13	0	27	6	21	35	27	31	0	17	0	3	43	1	1	12	3
	riparian	3	0	7	0	0	0	0	8	6	6	0	0	0	0	5	0	1	6	0
Young forest (1-7 y post-fire)	heath	0	0	0	0	0	0	0	0	12	8	13	0	18	3	0	0	4	0	0
	eucalypt	22	15	51	10	64	53	0	1	17	16	27	10	14	19	6	8	19	4	2
	riparian	2	8	23	1	0	0	0	0	4	3	5	0	4	1	0	0	2	1	0
Recently burnt forest (<1 y post-fire)	heath	0	0	0	0	0	0	2[a]	0	0	1	14	3[a]	10	4	0	1	4	2	1
	eucalypt	0	0	0	0	0	0	27[a]	0	6	4	0	6[a]	0	1	0	0	4	6	1
	riparian	0	0	0	0	0	0	0	0	0	0	1	0	0	1	0	0	1	2	0
	edge (forest)	0	1	1	0	0	0	1	6	5	5	4	2	7	6	3	3	9	4	5
Other	edge (open)	0	0	0	0	0	0	0	3	2	3	4	1	7	5	3	2	8	3	6
	plantation	0	0	0	0	0	0	3	0	0	0	0	13	0	0	0	23	0	6	12
	reservoir	0	0	0	1	0	4	0	2	2	2	0	1	1	0	2	1	1	2	6
	farm stream	0	0	0	0	0	0	0	0	0	0	3	0	5	3	2	1	5	2	2
	farmland	0	0	0	0	0	0	0	7	3	3	8	4	16	16	30	11	22	29	41
	road	3	4	4	5	8	7	10	6	7	8	6	8	6	11	4	7	9	8	5
	urban	0	0	0	0	0	0	0	0	3	3	0	0	1	2	0	0	4	1	1

[a] a prescribed burn was conducted within these animals' available habitat during the study, converting a proportion of the heathy and eucalypt forest from >35 year to <1 year post-fire. Proportions relate to post-fire availability.

Supplementary Table S2. [excel spreadsheet]

Supplementary Table S3. [excel spreadsheet]

Supplementary Table S4. Odds ratio matrices for fine-scale habitat selection analysis by individual red fox *Vulpes vulpes* during day and night. Habitat types are listed in order from least to most strongly selected habitat. Values in the matrix of habitat types indicate the estimated odds ratio (LCL, UCL) for the habitat type in the row relative to the habitat type in the column. An odds ratio of 1 indicates equal selection, values < 1 indicate that the habitat type in the column is selected less than the habitat in the row, values >1 indicated that the habitat type in the column is more strongly selected. * indicates where the confidence limits do not overlap one.

Fox DUST

day ($n_{observed}$ = 123)

variable	avail.	Riparian (>35y)	Reservoir/dam	Eucalypt (>35y)	Road	Edge (forest)
Riparian (>35y)	0.20	NA	0.7 (0.1, 3.4)	0.4 (0.2, 0.8)*	0.3 (0.1, 0.9)*	0.1 (0, 0.4)*
Reservoir/dam	0.02	1.4 (0.3, 6.8)	NA	0.6 (0.1, 2.6)	0.5 (0.1, 2.4)	0.2 (0, 1.2)
Eucalypt (>35y)	0.71	2.4 (1.3, 4.5)*	1.7 (0.4, 7.3)	NA	0.8 (0.4, 1.7)	0.4 (0.1, 0.9)*
Road	0.05	3 (1.2, 7.7)*	2.1 (0.4, 10.7)	1.2 (0.6, 2.7)	NA	0.4 (0.1, 1.4)
Edge (forest)	0.02	6.8 (2.2, 20.5)*	4.7 (0.9, 26.3)	2.8 (1.1, 7.3)*	2.3 (0.7, 7.2)	NA

night ($n_{observed}$ = 93)

variable	avail.	Riparian (>35y)	Eucalypt (>35y)	Reservoir/dam	Edge (forest)	Road
Riparian (>35y)	0.19	NA	0.8 (0.4, 1.5)	0.5 (0.2, 1.2)	0.4 (0.1, 1.4)	0.4 (0.2, 1)
Eucalypt (>35y)	0.66	1.2 (0.7, 2.3)	NA	0.6 (0.2, 1.3)	0.5 (0.2, 1.6)	0.5 (0.2, 1.1)
Reservoir/dam	0.06	2.2 (0.9, 5.7)	1.8 (0.8, 4.2)	NA	0.9 (0.2, 3.6)	0.9 (0.3, 2.6)
Edge (forest)	0.03	2.4 (0.7, 8)	1.9 (0.6, 6)	1.1 (0.3, 4.1)	NA	1.0 (0.3, 3.5)
Road	0.07	2.4 (1, 5.7)	2 (0.9, 4.1)	1.1 (0.4, 3.1)	1.0 (0.3, 3.5)	NA

Fox 4780

day ($n_{observed}$ = 285)

variable	avail.	Eucalypt (>35y)	Road	Eucalypt (7-35y)	Eucalypt (1-7y)	Reservoir/dam
Eucalypt (>35y)	0.07	NA	0.3 (0.1, 1)	0.2 (0, 0.8)*	0.1 (0, 0.5)*	0 (0, 0.1)*
Road	0.05	3.6 (1, 13.9)	NA	0.7 (0.2, 2.1)	0.5 (0.3, 1.1)	0.1 (0.1, 0.3)*
Eucalypt (7-35y)	0.03	5.2 (1.3, 21.4)*	1.4 (0.5, 4.2)	NA	0.8 (0.3, 1.8)	0.2 (0.1, 0.5)*
Eucalypt (1-7y)	0.76	6.8 (2.1, 21.6)*	1.9 (0.9, 3.9)	1.3 (0.6, 3)	NA	0.3 (0.2, 0.4)*
Reservoir/dam	0.1	24.9 (7.6, 82.1)*	6.9 (3.1, 15.1)*	4.8 (2, 11.7)*	3.7 (2.5, 5.3)*	NA

night ($n_{observed}$ = 561)

variable	avail.	Eucalypt (>35y)	Eucalypt (7-35y)	Road	Eucalypt (1-7y)	Reservoir/dam
Eucalypt (>35y)	0.08	NA	0.3 (0, 1.7)	0.1 (0, 0.2)*	0 (0, 0.2)*	0 (0, 0.1)*
Eucalypt (7-35y)	0.03	3.6 (0.6, 21.9)	NA	0.2 (0.1, 0.6)*	0.2 (0.1, 0.5)*	0.1 (0, 0.2)*
Road	0.06	19 (4.5, 80.3)*	5.3 (1.6, 17.5)*	NA	0.8 (0.6, 1.2)	0.3 (0.2, 0.5)*
Eucalypt (1-7y)	0.71	22.7 (5.6, 91.8)*	6.3 (2, 19.9)*	1.2 (0.8, 1.8)	NA	0.4 (0.3, 0.5)*
Reservoir/dam	0.12	61.7 (15.1, 252.4)*	17.1 (5.3, 55)*	3.2 (2.1, 5.1)*	2.7 (2.1, 3.5)*	NA

Fox 4190

day (pre-fire) ($n_{observed}$ = 217)

variable	avail.	Eucalypt (7-35y)	Road	Eucalypt (>35y)	Eucalypt (will be <1y)
Eucalypt (7-35y)	0.09	NA	0.2 (0.1, 0.6)*	0.2 (0.1, 0.5)*	0.1 (0, 0.4)*
Road	0.12	5.3 (1.8, 15.5)*	NA	0.9 (0.6, 1.5)	0.7 (0.4, 1.2)
Eucalypt (>35y)	0.44	5.9 (2.1, 16.3)*	1.1 (0.7, 1.8)	NA	0.8 (0.5, 1.2)
Eucalypt (will be <1y)	0.34	7.4 (2.7, 20.8)*	1.4 (0.9, 2.3)	1.3 (0.9, 1.9)	NA

day (post-fire) ($n_{observed}$ = 42)

variable	avail.	Road	Eucalypt (<1y)	Eucalypt (>35y)
Eucalypt (7-35y)[a]	0.10			
Road	0.13	NA	0.5 (0.1, 1.8)	0.4 (0.1, 1.6)
Eucalypt (<1y)	0.53	1.9 (0.6, 6.8)	NA	0.9 (0.4, 2)
Eucalypt (>35y)	0.34	2.2 (0.6, 8)	1.1 (0.5, 2.6)	NA

[a] This habitat was available but never used post-fire and therefore had to be dropped from the analysis

night (pre-fire) ($n_{observed}$ = 320)

variable	avail.	Eucalypt (7-35y)	Eucalypt (>35y)	Eucalypt (will be <1y)	Road
Eucalypt (7-35y)	0.15	NA	0.4 (0.2, 0.7)*	0.4 (0.2, 0.6)*	0.2 (0.1, 0.3)*
Eucalypt (>35y)	0.31	2.5 (1.4, 4.2)*	NA	0.9 (0.6, 1.3)	0.4 (0.3, 0.6)*
Eucalypt (will be <1y)	0.38	2.8 (1.6, 4.7)*	1.1 (0.8, 1.6)	NA	0.5 (0.3, 0.6)*
Road	0.15	5.8 (3.5, 9.7)*	2.4 (1.7, 3.3)*	2.1 (1.6, 2.9)*	NA

night (post-fire) ($n_{observed}$ = 82)

variable	avail.	Eucalypt (7-35y)	Eucalypt (>35y)	Eucalypt (<1y)	Road
Eucalypt (7-35y)	0.16	NA	0.3 (0.1, 1.2)	0.2 (0, 0.5)*	0.1 (0, 0.4)*
Eucalypt (>35y)	0.12	3.8 (0.8, 17.6)	NA	0.6 (0.2, 1.7)	0.4 (0.1, 1.2)
Eucalypt (<1y)	0.59	6.4 (1.9, 22.4)*	1.7 (0.6, 5)	NA	0.7 (0.4, 1.3)
Road	0.14	9.1 (2.6, 32.1)*	2.4 (0.8, 7)	1.4 (0.8, 2.5)	NA

Fox GULY

day ($n_{observed}$ = 4) – individual analysis was not conducted due to small sample size

night ($n_{observed}$ =15) – individual analysis was not conducted due to small sample size

Fox SAND

day ($n_{observed}$ = 307)

variable	avail.	Heath (1-7y)	Riparian (1-7y)	Eucalypt (1-7y)	Eucalypt (7-35y)	Road	Edge (forest)	Heath (7-35y)	Riparian (7-35y)
Heath (1-7y)	0.07	NA	0.8 (0.4, 1.7)	0.7 (0.4, 1.3)	0.7 (0.4, 1.2)	0.7 (0.3, 1.3)	0.5 (0.3, 1.1)	0.4 (0.2, 0.9)*	0.4 (0.2, 0.8)*
Riparian (1-7y)	0.06	1.3 (0.6, 2.7)	NA	0.9 (0.5, 1.6)	0.8 (0.5, 1.5)	0.8 (0.4, 1.6)	0.7 (0.3, 1.4)	0.6 (0.3, 1)	0.5 (0.2, 1)*
Eucalypt (1-7y)	0.26	1.4 (0.8, 2.6)	1.1 (0.6, 2)	NA	0.9 (0.7, 1.4)	0.9 (0.6, 1.5)	0.8 (0.4, 1.3)	0.6 (0.4, 1)*	0.5 (0.3, 0.9)*
Eucalypt (7-35y)	0.25	1.5 (0.8, 2.8)	1.2 (0.7, 2.2)	1.1 (0.7, 1.5)	NA	1.0 (0.6, 1.6)	0.8 (0.5, 1.4)	0.7 (0.5, 1)	0.6 (0.3, 1)*
Road	0.08	1.5 (0.8, 3)	1.2 (0.6, 2.3)	1.1 (0.7, 1.7)	1.0 (0.6, 1.6)	NA	0.8 (0.4, 1.5)	0.7 (0.4, 1.2)	0.6 (0.3, 1.1)
Edge (forest)	0.05	1.9 (0.9, 3.8)	1.5 (0.7, 3)	1.3 (0.8, 2.3)	1.2 (0.7, 2.1)	1.2 (0.7, 2.3)	NA	0.8 (0.5, 1.5)	0.7 (0.4, 1.4)
Heath (7-35y)	0.17	2.2 (1.2, 4.3)*	1.8 (1, 3.3)	1.6 (1, 2.4)*	1.5 (1, 2.2)	1.5 (0.9, 2.5)	1.2 (0.7, 2.1)	NA	0.9 (0.5, 1.5)
Riparian (7-35y)	0.05	2.6 (1.3, 5.4)*	2.1 (1, 4.1)*	1.8 (1.1, 3.1)*	1.7 (1, 2.9)*	1.7 (0.9, 3.2)	1.4 (0.7, 2.7)	1.2 (0.7, 2)	NA

night ($n_{observed}$ = 338)

variable	avail.	Eucalypt (1-7y)	Heath (1-7y)	Heath (7-35y)	Riparian (7-35y)	Eucalypt (7-35y)	Edge (forest)	Riparian (1-7y)	Farmland	Edge (open)	Road
Eucalypt (1-7y)	0.19	NA	0.6 (0.3, 1.4)	0.5 (0.3, 1)*	0.4 (0.2, 0.9)*	0.3 (0.2, 0.5)*	0.3 (0.2, 0.6)*	0.3 (0.1, 0.5)*	0.2 (0.1, 0.5)*	0.2 (0.1, 0.4)*	0.1 (0.1, 0.2)*
Heath (1-7y)	0.06	1.6 (0.7, 3.4)	NA	0.8 (0.4, 1.8)	0.7 (0.3, 1.6)	0.5 (0.2, 1)*	0.5 (0.2, 1.1)	0.4 (0.2, 1)*	0.4 (0.2, 0.9)*	0.3 (0.1, 0.8)*	0.1 (0.1, 0.3)*
Heath (7-35y)	0.1	2 (1, 3.7)*	1.3 (0.6, 2.8)	NA	0.8 (0.4, 1.8)	0.6 (0.4, 1)	0.6 (0.3, 1.2)	0.5 (0.3, 1.1)	0.5 (0.2, 0.9)*	0.4 (0.2, 0.9)*	0.2 (0.1, 0.3)*
Riparian (7-35y)	0.04	2.4 (1.1, 5.3)*	1.5 (0.6, 3.9)	1.2 (0.6, 2.7)	NA	0.7 (0.4, 1.5)	0.7 (0.3, 1.6)	0.7 (0.3, 1.5)	0.6 (0.3, 1.3)	0.5 (0.2, 1.2)	0.2 (0.1, 0.4)*
Eucalypt (7-35y)	0.2	3.2 (1.9, 5.4)*	2.1 (1, 4.3)*	1.7 (1, 2.8)	1.3 (0.7, 2.7)	NA	1.0 (0.6, 1.7)	0.9 (0.5, 1.6)	0.8 (0.4, 1.4)	0.7 (0.4, 1.3)	0.3 (0.2, 0.4)*
Edge (forest)	0.08	3.3 (1.7, 6.1)*	2.1 (0.9, 4.7)	1.7 (0.9, 3.2)	1.4 (0.6, 3)	1.0 (0.6, 1.7)	NA	0.9 (0.5, 1.8)	0.8 (0.4, 1.5)	0.7 (0.4, 1.4)	0.3 (0.2, 0.5)*
Riparian (1-7y)	0.05	3.6 (1.9, 6.9)*	2.3 (1, 5.2)*	1.8 (0.9, 3.6)	1.5 (0.7, 3.4)	1.1 (0.6, 2)	1.1 (0.6, 2.2)	NA	0.9 (0.4, 1.8)	0.8 (0.4, 1.6)	0.3 (0.2, 0.6)*
Farmland	0.09	4.2 (2.1, 8.2)*	2.7 (1.2, 6.2)*	2.1 (1.1, 4.3)*	1.8 (0.8, 3.9)	1.3 (0.7, 2.3)	1.3 (0.7, 2.4)	1.2 (0.6, 2.4)	NA	0.9 (0.5, 1.8)	0.4 (0.2, 0.7)*
Edge (open)	0.05	4.5 (2.3, 8.9)*	2.9 (1.3, 6.7)*	2.3 (1.2, 4.6)*	1.9 (0.8, 4.3)	1.4 (0.8, 2.5)	1.4 (0.7, 2.7)	1.3 (0.6, 2.6)	1.1 (0.6, 2.1)	NA	0.4 (0.3, 0.7)*
Road	0.14	10.7 (6.6, 17.3)*	6.8 (3.4, 13.6)*	5.4 (3.3, 9.1)*	4.4 (2.2, 8.8)*	3.3 (2.3, 4.7)*	3.3 (2, 5.3)*	3 (1.7, 5.2)*	2.5 (1.5, 4.3)*	2.3 (1.4, 4)*	NA

Fox REED

day ($n_{observed}$ = 3) – individual analysis was not conducted due to small sample size

night ($n_{observed}$ = 28) – individual analysis was not conducted due to small sample size

Fox CINN

day ($n_{observed}$ = 97)

variable	avail.	Riparian (>35y)	Eucalypt (1-7y)	Riparian (1-7y)	Eucalypt (>35y)	Road	Farmland	Heath (<1y)	Heath (1-7y)	Heath (7-35y)	Edge (open)	Edge (forest)
Riparian (>35y)	0.02	NA	0.7 (0.1, 5.4)	0.5 (0, 5)	0.5 (0.1, 4.6)	0.4 (0, 3.7)	0.3 (0, 2.9)	0.3 (0, 2.4)	0.3 (0, 2.1)	0.3 (0, 2.3)	0.2 (0, 2.2)	0.2 (0, 1.9)
Eucalypt (1-7y)	0.26	1.4 (0.2, 11.3)	NA	0.7 (0.2, 2.6)	0.7 (0.2, 2.2)	0.6 (0.2, 1.6)	0.4 (0.1, 1.4)	0.4 (0.2, 1)*	0.4 (0.2, 0.8)*	0.4 (0.1, 1.1)	0.3 (0.1, 1.2)	0.3 (0.1, 0.9)*
Riparian (1-7y)	0.04	2 (0.2, 20.4)	1.4 (0.4, 5.2)	NA	1.0 (0.2, 4.7)	0.8 (0.2, 3.6)	0.6 (0.1, 2.9)	0.6 (0.1, 2.2)	0.5 (0.1, 1.9)	0.5 (0.1, 2.3)	0.4 (0.1, 2.3)	0.4 (0.1, 1.9)
Eucalypt (>35y)	0.04	2.1 (0.2, 19.4)	1.4 (0.4, 4.6)	1.0 (0.2, 4.8)	NA	0.8 (0.2, 3.4)	0.6 (0.1, 2.7)	0.6 (0.2, 2.1)	0.5 (0.2, 1.8)	0.5 (0.1, 2.1)	0.4 (0.1, 2.2)	0.4 (0.1, 1.8)
Road	0.07	2.4 (0.3, 21.8)	1.7 (0.6, 4.7)	1.2 (0.3, 5.2)	1.2 (0.3, 4.8)	NA	0.8 (0.2, 2.7)	0.7 (0.3, 1.9)	0.6 (0.2, 1.6)	0.6 (0.2, 2)	0.5 (0.1, 2.2)	0.5 (0.1, 1.8)
Farmland	0.05	3.2 (0.3, 30.4)	2.2 (0.7, 7)	1.6 (0.4, 7.2)	1.6 (0.4, 6.7)	1.3 (0.4, 4.7)	NA	0.9 (0.3, 2.7)	0.8 (0.3, 2.3)	0.8 (0.2, 2.7)	0.6 (0.1, 2.9)	0.6 (0.2, 2.5)
Heath (<1y)	0.13	3.5 (0.4, 29.8)	2.5 (1, 5.9)*	1.7 (0.4, 6.8)	1.7 (0.5, 6.2)	1.4 (0.5, 4)	1.1 (0.4, 3.3)	NA	0.9 (0.4, 1.8)	0.9 (0.3, 2.2)	0.7 (0.2, 2.8)	0.7 (0.2, 2.3)
Heath (1-7y)	0.26	3.9 (0.5, 31.8)	2.7 (1.3, 5.9)*	1.9 (0.5, 7.2)	1.9 (0.6, 6.5)	1.6 (0.6, 4.1)	1.2 (0.4, 3.5)	1.1 (0.5, 2.3)	NA	1.0 (0.4, 2.5)	0.8 (0.2, 2.9)	0.8 (0.2, 2.4)
Heath (7-35y)	0.07	4 (0.4, 36.6)	2.8 (0.9, 8.2)	2 (0.4, 8.8)	1.9 (0.5, 8)	1.6 (0.5, 5.3)	1.2 (0.4, 4.1)	1.1 (0.4, 2.9)	1.0 (0.4, 2.6)	NA	0.8 (0.2, 3.4)	0.8 (0.2, 2.9)
Edge (open)	0.02	5 (0.5, 54.3)	3.5 (0.9, 14.1)	2.5 (0.4, 13.6)	2.4 (0.5, 12.9)	2 (0.5, 9.1)	1.5 (0.3, 6.9)	1.4 (0.4, 5.4)	1.3 (0.3, 4.7)	1.2 (0.3, 5.3)	NA	1.0 (0.2, 4.6)
Edge (forest)	0.03	5.2 (0.5, 51.4)	3.6 (1.1, 12.1)*	2.6 (0.5, 12.8)	2.5 (0.6, 11.5)	2.1 (0.6, 8.1)	1.6 (0.4, 6.4)	1.5 (0.4, 4.9)	1.3 (0.4, 4.2)	1.3 (0.3, 4.9)	1.0 (0.2, 5)	NA

night ($n_{observed}$ = 320)

variable	avail.	Eucalypt (>35y)	Riparian (1-7y)	Riparian (>35y)	Heath (>35y)	Eucalypt (1-7y)	Road	Heath (<1y)	Heath (7-35y)	Farmland	Edge (forest)	Heath (1-7y)	Edge (open)	Farm-stream
Eucalypt (>35y)	0.06	NA	0.9 (0.2, 5.1)	0.6 (0.1, 2.6)	0.4 (0.1, 1.6)	0.2 (0.1, 0.7)*	0.2 (0.1, 0.7)*	0.2 (0.1, 0.6)*	0.2 (0, 0.6)*	0.1 (0, 0.4)*	0.1 (0, 0.4)*	0.1 (0, 0.3)*	0.1 (0, 0.2)*	0 (0, 0.1)*
Riparian (1-7y)	0.03	1.1 (0.2, 6.1)	NA	0.6 (0.1, 3.8)	0.4 (0.1, 2.4)	0.3 (0.1, 1.1)	0.2 (0, 1)	0.2 (0, 0.9)*	0.2 (0, 0.9)*	0.2 (0, 0.7)*	0.1 (0, 0.6)*	0.1 (0, 0.4)*	0.1 (0, 0.3)*	0 (0, 0.2)*
Riparian (>35y)	0.03	1.8 (0.4, 8.1)	1.6 (0.3, 9.9)	NA	0.6 (0.1, 3.2)	0.4 (0.1, 1.4)	0.4 (0.1, 1.3)	0.3 (0.1, 1.2)	0.3 (0.1, 1.2)	0.3 (0.1, 0.9)*	0.2 (0.1, 0.8)*	0.2 (0, 0.5)*	0.1 (0, 0.4)*	0.1 (0, 0.3)*
Heath (>35y)	0.02	2.8 (0.6, 12.4)	2.6 (0.4, 15.7)	1.6 (0.3, 8.1)	NA	0.7 (0.2, 2.2)	0.6 (0.2, 2)	0.5 (0.2, 1.9)	0.5 (0.1, 1.9)	0.4 (0.1, 1.3)	0.4 (0.1, 1.2)	0.3 (0.1, 0.8)*	0.2 (0.1, 0.6)*	0.1 (0, 0.4)*
Eucalypt (1-7y)	0.19	4.1 (1.4, 11.7)*	3.8 (0.9, 15.8)	2.3 (0.7, 7.7)	1.5 (0.4, 4.8)	NA	0.8 (0.4, 1.6)	0.8 (0.4, 1.5)	0.7 (0.3, 1.6)	0.6 (0.4, 1)*	0.5 (0.3, 0.9)*	0.4 (0.2, 0.6)*	0.3 (0.2, 0.4)*	0.2 (0.1, 0.3)*
Road	0.06	4.8 (1.5, 15.3)*	4.4 (1, 20)	2.7 (0.8, 10)	1.7 (0.5, 6.1)	1.2 (0.6, 2.2)	NA	0.9 (0.5, 1.9)	0.8 (0.3, 2)	0.7 (0.4, 1.3)	0.6 (0.3, 1.2)	0.4 (0.2, 0.8)*	0.3 (0.2, 0.6)*	0.2 (0.1, 0.4)*
Heath (<1y)	0.08	5.1 (1.6, 15.9)*	4.7 (1.1, 20.8)*	2.9 (0.8, 10.4)	1.8 (0.5, 6.3)	1.2 (0.7, 2.3)	1.1 (0.5, 2.2)	NA	0.9 (0.4, 2)	0.7 (0.4, 1.4)	0.7 (0.4, 1.3)	0.5 (0.3, 0.8)*	0.3 (0.2, 0.6)*	0.2 (0.1, 0.4)*
Heath (7-35y)	0.03	5.8 (1.7, 20.5)*	5.4 (1.1, 26.1)*	3.3 (0.8, 13.2)	2.1 (0.5, 8)	1.4 (0.6, 3.2)	1.2 (0.5, 2.9)	1.1 (0.5, 2.6)	NA	0.8 (0.4, 1.9)	0.8 (0.3, 1.7)	0.5 (0.2, 1.2)	0.4 (0.2, 0.8)*	0.3 (0.1, 0.6)*
Farmland	0.12	7 (2.4, 20.5)*	6.4 (1.5, 27.5)*	4 (1.2, 13.5)*	2.5 (0.8, 8.2)	1.7 (1, 2.8)*	1.5 (0.8, 2.8)	1.4 (0.7, 2.6)	1.2 (0.5, 2.7)	NA	0.9 (0.6, 1.5)	0.6 (0.4, 1.1)	0.4 (0.3, 0.7)*	0.3 (0.2, 0.5)*
Edge (forest)	0.09	7.7 (2.6, 22.6)*	7.1 (1.6, 30.3)*	4.4 (1.3, 14.9)*	2.7 (0.8, 9)	1.9 (1.1, 3.2)*	1.6 (0.8, 3.1)	1.5 (0.8, 2.9)	1.3 (0.6, 3)	1.1 (0.7, 1.8)	NA	0.7 (0.4, 1.2)	0.5 (0.3, 0.7)*	0.3 (0.2, 0.6)*
Heath (1-7y)	0.14	11 (3.8, 31.8)*	10.1 (2.4, 42.4)*	6.2 (1.8, 21)*	3.9 (1.2, 12.8)*	2.7 (1.7, 4.2)*	2.3 (1.2, 4.2)*	2.1 (1.2, 3.8)*	1.9 (0.9, 4.1)	1.6 (1, 2.6)	1.4 (0.9, 2.4)	NA	0.7 (0.4, 1.1)	0.5 (0.3, 0.8)*
Edge (open)	0.09	16.3 (5.7, 46.7)*	14.9 (3.6, 62.9)*	9.2 (2.8, 30.8)*	5.8 (1.8, 18.7)*	4 (2.5, 6.3)*	3.4 (1.8, 6.3)*	3.2 (1.8, 5.8)*	2.8 (1.3, 6.2)*	2.3 (1.5, 3.6)*	2.1 (1.3, 3.3)*	1.5 (0.9, 2.3)	NA	0.7 (0.5, 1.2)
Farm-stream	0.04	22.3 (7.5, 66.4)*	20.4 (4.7, 88.3)*	12.6 (3.6, 43.7)*	7.9 (2.4, 26.4)*	5.4 (3.1, 9.3)*	4.6 (2.3, 9.2)*	4.4 (2.3, 8.4)*	3.8 (1.7, 8.8)*	3.2 (1.9, 5.3)*	2.9 (1.7, 5)*	2 (1.2, 3.5)*	1.4 (0.8, 2.2)	NA

Fox 6410

day (pre-fire) ($n_{observed}$ = 228)

variable	avail.	Eucalypt (7-35y)	Eucalypt (will be <1y)	Road	Plantation	Eucalypt (>35y)	Heath (will be <1y)
Eucalypt (7-35y)	0.22	NA	1.0 (0.6, 1.6)	0.7 (0.4, 1.1)	0.6 (0.3, 1.6)	0.6 (0.3, 1)	0.5 (0.3, 0.8)*
Eucalypt (will be <1y)	0.34	1.0 (0.6, 1.6)	NA	0.7 (0.4, 1.1)	0.6 (0.3, 1.5)	0.6 (0.3, 1)*	0.5 (0.3, 0.8)*
Road	0.13	1.5 (0.9, 2.4)	1.4 (0.9, 2.3)	NA	0.9 (0.4, 2.2)	0.8 (0.5, 1.5)	0.7 (0.4, 1.2)
Plantation	0.03	1.6 (0.6, 3.9)	1.6 (0.7, 3.6)	1.1 (0.5, 2.6)	NA	0.9 (0.4, 2.2)	0.8 (0.3, 1.8)
Eucalypt (>35y)	0.07	1.7 (1, 3.1)	1.7 (1, 2.9)*	1.2 (0.7, 2.1)	1.1 (0.4, 2.7)	NA	0.8 (0.5, 1.5)
Heath (will be <1y)	0.20	2.1 (1.2, 3.6)*	2 (1.3, 3.3)*	1.4 (0.9, 2.4)	1.3 (0.6, 3.1)	1.2 (0.7, 2.1)	NA

day (post-fire) ($n_{observed}$ = 12) – analysis was not conducted due to small sample size

Fox 6410 [cont]

night (pre-fire) ($n_{observed}$ = 432)

variable	avail.	Eucalypt (>35y)	Eucalypt (7-35y)	Plantation	Eucalypt (will be <1y)	Heath (will be <1y)	Edge (forest)	Farmland	Road
Eucalypt (>35y)	0.09	NA	0.8 (0.4, 1.7)	0.6 (0.3, 1.2)	0.4 (0.2, 0.7)*	0.3 (0.1, 0.6)*	0.2 (0.1, 0.5)*	0.2 (0.1, 0.4)*	0.2 (0.1, 0.3)*
Eucalypt (7-35y)	0.09	1.3 (0.6, 2.7)	NA	0.7 (0.3, 1.4)	0.5 (0.3, 0.8)*	0.4 (0.2, 0.8)*	0.3 (0.1, 0.7)*	0.2 (0.1, 0.5)*	0.2 (0.1, 0.4)*
Plantation	0.07	1.8 (0.9, 3.8)	1.4 (0.7, 3)	NA	0.6 (0.4, 1.1)	0.6 (0.3, 1.1)	0.4 (0.2, 0.9)*	0.4 (0.2, 0.7)*	0.3 (0.2, 0.5)*
Eucalypt (will be <1y)	0.31	2.8 (1.5, 5.1)*	2.2 (1.3, 3.8)*	1.5 (0.9, 2.7)	NA	0.9 (0.5, 1.4)	0.6 (0.3, 1.3)	0.5 (0.3, 1)	0.5 (0.3, 0.6)*
Heath (will be <1y)	0.07	3.3 (1.6, 6.8)*	2.6 (1.3, 5.1)*	1.8 (0.9, 3.5)	1.2 (0.7, 1.9)	NA	0.7 (0.3, 1.7)	0.6 (0.3, 1.4)	0.5 (0.3, 0.9)*
Edge (forest)	0.03	4.5 (1.9, 10.3)*	3.5 (1.5, 8.4)*	2.5 (1.1, 5.5)*	1.6 (0.8, 3.3)	1.4 (0.6, 3.1)	NA	0.9 (0.5, 1.6)	0.7 (0.4, 1.5)
Farmland	0.27	5.2 (2.5, 10.8)*	4.1 (1.9, 9)*	2.9 (1.4, 5.9)*	1.9 (1, 3.5)	1.6 (0.7, 3.4)	1.2 (0.6, 2.2)	NA	0.8 (0.4, 1.6)
Road	0.09	6.1 (3.3, 11.3)*	4.8 (2.8, 8.4)*	3.4 (1.9, 5.9)*	2.2 (1.6, 3)*	1.9 (1.1, 3.1)*	1.4 (0.7, 2.8)	1.2 (0.6, 2.2)	NA

night (post-fire) ($n_{observed}$ = 32)

variable	avail.	Eucalypt (7-35y)	Eucalypt (>35y)	Eucalypt (<1y)	Heath (<1y)	Road
Plantation[a]	0.03					
Heath (>35y)[a]	0.03					
Eucalypt (7-35y)	0.15	NA	0.4 (0, 4.6)	0.3 (0.1, 1.6)	0.2 (0, 1.2)	0.2 (0, 1.2)
Eucalypt (>35y)	0.04	2.8 (0.2, 35.5)	NA	0.8 (0.1, 6.6)	0.5 (0.1, 4.7)	0.5 (0.1, 4.8)
Eucalypt (<1y)	0.42	3.5 (0.6, 19.5)	1.3 (0.2, 10.9)	NA	0.7 (0.2, 2.1)	0.7 (0.2, 2.2)
Heath (<1y)	0.28	5.1 (0.8, 31.9)	1.9 (0.2, 16.3)	1.4 (0.5, 4.3)	NA	1.0 (0.3, 3.2)
Road	0.11	5.2 (0.8, 31.9)	1.9 (0.2, 16.8)	1.5 (0.5, 4.6)	1.0 (0.3, 3.3)	NA

[a] These habitat types were available but never used post-fire and therefore had to be dropped from the analysis

Fox RUSH

day ($n_{observed}$ = 131)

variable	avail.	Road	Eucalypt (>35y)	Eucalypt (1-7y)	Riparian (1-7y)	Heath (<1y)	Heath (1-7y)	Heath (7-35y)	Edge (forest)	Farm-stream	Farmland	Edge (open)
Riparian (>35y) [a]	0.02											
Heath (>35y) [a]	0.03											
Road	0.06	NA	1.0 (0.2, 5.2)	0.9 (0.2, 3.5)	0.6 (0.1, 3.4)	0.5 (0.1, 1.9)	0.3 (0.1, 1)	0.3 (0.1, 1)	0.2 (0.1, 0.9)*	0.2 (0.1, 0.9)*	0.2 (0.1, 0.7)*	0.2 (0.1, 0.7)*
Eucalypt (>35y)	0.05	1.0 (0.2, 5.7)	NA	1.0 (0.2, 4)	0.7 (0.1, 3.7)	0.5 (0.1, 2.2)	0.3 (0.1, 1.2)	0.3 (0.1, 1.2)	0.3 (0.1, 1)*	0.2 (0.1, 1)*	0.2 (0.1, 0.8)*	0.2 (0.1, 0.8)*
Eucalypt (1-7y)	0.18	1.1 (0.3, 4)	1.0 (0.3, 4.1)	NA	0.7 (0.2, 2.6)	0.5 (0.2, 1.4)	0.3 (0.1, 0.7)*	0.3 (0.1, 0.8)*	0.3 (0.1, 0.6)*	0.2 (0.1, 0.7)*	0.2 (0.1, 0.5)*	0.2 (0.1, 0.5)*
Riparian (1-7y)	0.04	1.5 (0.3, 8.2)	1.5 (0.3, 8.1)	1.5 (0.4, 5.4)	NA	0.8 (0.2, 3)	0.5 (0.1, 1.7)	0.4 (0.1, 1.7)	0.4 (0.1, 1.4)	0.3 (0.1, 1.4)	0.3 (0.1, 1.1)	0.3 (0.1, 1.1)
Heath (<1y)	0.11	2 (0.5, 7.5)	1.9 (0.5, 7.9)	1.9 (0.7, 4.9)	1.3 (0.3, 5.1)	NA	0.6 (0.3, 1.3)	0.5 (0.2, 1.4)	0.5 (0.2, 1.2)	0.5 (0.2, 1.2)	0.4 (0.2, 0.9)*	0.4 (0.2, 0.9)*
Heath (1-7y)	0.15	3.3 (1, 11.5)	3.2 (0.8, 12.3)	3.1 (1.4, 7)*	2.2 (0.6, 7.8)	1.7 (0.7, 3.7)	NA	0.9 (0.4, 2.2)	0.8 (0.4, 1.8)	0.8 (0.3, 1.9)	0.7 (0.3, 1.3)	0.7 (0.3, 1.4)
Heath (7-35y)	0.06	3.8 (1, 14.6)	3.6 (0.8, 15.3)	3.5 (1.3, 9.6)*	2.4 (0.6, 9.8)	1.9 (0.7, 4.7)	1.1 (0.5, 2.7)	NA	0.9 (0.4, 2.3)	0.8 (0.3, 2.4)	0.7 (0.3, 1.7)	0.7 (0.3, 1.8)
Edge (forest)	0.09	4 (1.1, 14.5)*	3.8 (1, 14.5)*	3.8 (1.6, 8.7)*	2.6 (0.7, 9.4)	2 (0.9, 4.8)	1.2 (0.6, 2.6)	1.1 (0.4, 2.7)	NA	0.9 (0.4, 2.3)	0.8 (0.4, 1.6)	0.8 (0.4, 1.6)
Farm-stream	0.05	4.4 (1.1, 17.7)*	4.2 (1, 17.9)*	4.2 (1.5, 11.6)*	2.9 (0.7, 11.8)	2.2 (0.8, 6.1)	1.3 (0.5, 3.4)	1.2 (0.4, 3.3)	1.1 (0.4, 2.8)	NA	0.9 (0.4, 2)	0.9 (0.3, 2.1)
Farmland	0.13	5.1 (1.5, 17.5)*	4.8 (1.3, 17.7)*	4.7 (2.2, 10.4)*	3.3 (0.9, 11.5)	2.5 (1.1, 5.6)*	1.5 (0.8, 3)	1.3 (0.6, 3.1)	1.3 (0.6, 2.5)	1.1 (0.5, 2.6)	NA	1.0 (0.5, 1.9)
Edge (open)	0.08	5.1 (1.4, 18.5)*	4.9 (1.3, 18.7)*	4.8 (2.1, 11.1)*	3.3 (0.9, 12)	2.6 (1.1, 6.1)*	1.5 (0.7, 3.3)	1.4 (0.6, 3.4)	1.3 (0.6, 2.7)	1.2 (0.5, 2.9)	1.0 (0.5, 1.9)	NA

[a] These habitats were available but never used and therefore had to be dropped from the analysis

night ($n_{observed}$ = 290)

variable	avail.	Eucalypt (>35y)	Riparian (>35y)	Heath (7-35y)	Heath (<1y)	Road	Eucalypt (1-7y)	Farmland	Edge (forest)	Heath (1-7y)	Farm-stream	Edge (open)
Eucalypt (>35y)	0.12	NA	1.0 (0.3, 2.8)	0.5 (0.2, 1)	0.4 (0.2, 0.9)*	0.4 (0.2, 0.7)*	0.3 (0.2, 0.7)*	0.3 (0.1, 0.5)*	0.3 (0.1, 0.5)*	0.2 (0.1, 0.5)*	0.2 (0.1, 0.5)*	0.2 (0.1, 0.3)*
Riparian (>35y)	0.04	1.0 (0.4, 3)	NA	0.5 (0.2, 1.4)	0.4 (0.1, 1.2)	0.4 (0.1, 1.1)	0.4 (0.1, 1)*	0.3 (0.1, 0.7)*	0.3 (0.1, 0.7)*	0.2 (0.1, 0.7)*	0.2 (0.1, 0.7)*	0.2 (0.1, 0.4)*
Heath (7-35y)	0.07	2.2 (1, 4.9)	2.1 (0.7, 6.2)	NA	0.9 (0.4, 1.9)	0.8 (0.4, 1.7)	0.7 (0.4, 1.5)	0.6 (0.3, 1.1)	0.6 (0.3, 1.1)	0.5 (0.2, 1.1)	0.5 (0.2, 1)	0.3 (0.2, 0.7)*
Heath (<1y)	0.08	2.5 (1.1, 5.5)*	2.4 (0.8, 6.9)	1.1 (0.5, 2.4)	NA	0.9 (0.4, 1.9)	0.8 (0.4, 1.7)	0.6 (0.4, 1.1)	0.6 (0.3, 1.2)	0.6 (0.3, 1.1)	0.6 (0.3, 1.1)	0.4 (0.2, 0.7)*
Road	0.07	2.7 (1.3, 5.4)*	2.6 (0.9, 7.2)	1.2 (0.6, 2.6)	1.1 (0.5, 2.2)	NA	0.9 (0.5, 1.8)	0.7 (0.4, 1.2)	0.7 (0.4, 1.3)	0.6 (0.3, 1.2)	0.6 (0.3, 1.2)	0.4 (0.2, 0.8)*
Eucalypt (1-7y)	0.1	2.9 (1.4, 6)*	2.8 (1, 7.6)*	1.3 (0.7, 2.8)	1.2 (0.6, 2.3)	1.1 (0.6, 2.1)	NA	0.8 (0.4, 1.3)	0.7 (0.4, 1.3)	0.7 (0.4, 1.2)	0.7 (0.4, 1.2)	0.5 (0.3, 0.8)*
Farmland	0.2	3.9 (2.1, 7.3)*	3.7 (1.4, 9.8)*	1.8 (0.9, 3.4)	1.6 (0.9, 2.8)	1.4 (0.8, 2.6)	1.3 (0.8, 2.2)	NA	1.0 (0.6, 1.6)	0.9 (0.5, 1.6)	0.9 (0.6, 1.4)	0.6 (0.4, 0.9)*
Edge (forest)	0.08	3.9 (2, 7.8)*	3.7 (1.4, 10.2)*	1.8 (0.9, 3.6)	1.6 (0.8, 3)	1.5 (0.8, 2.7)	1.3 (0.8, 2.4)	1.0 (0.6, 1.6)	NA	0.9 (0.5, 1.7)	0.9 (0.5, 1.6)	0.6 (0.4, 1)
Heath (1-7y)	0.07	4.3 (2, 9.3)*	4.2 (1.5, 11.7)*	2 (0.9, 4.2)	1.8 (0.9, 3.5)	1.6 (0.8, 3.2)	1.5 (0.8, 2.7)	1.1 (0.6, 2)	1.1 (0.6, 2.1)	NA	1.0 (0.5, 1.9)	0.7 (0.4, 1.2)
Farm-stream	0.08	4.3 (2.2, 8.6)*	4.2 (1.5, 11.4)*	2 (1, 4)	1.8 (0.9, 3.4)	1.6 (0.9, 3)	1.5 (0.8, 2.7)	1.1 (0.7, 1.8)	1.1 (0.6, 1.9)	1.0 (0.5, 1.9)	NA	0.7 (0.4, 1.1)
Edge (open)	0.09	6.4 (3.3, 12.5)*	6.1 (2.3, 16.4)*	2.9 (1.5, 5.7)*	2.6 (1.4, 4.7)*	2.4 (1.3, 4.3)*	2.2 (1.3, 3.7)*	1.7 (1.1, 2.5)*	1.6 (1, 2.7)	1.5 (0.8, 2.6)	1.5 (0.9, 2.4)	NA

Fox RUST

day ($n_{observed}$ = 84)

variable	avail.	Eucalypt (>35y)	Road	Riparian (<1y)	Farm-stream	Heath (<1y)	Heath (>35y)	Farmland	Edge (open)	Heath (7-35y)	Riparian (>35y)	Edge (forest)
Eucalypt (<1y) [a]	0.06											
Eucalypt (>35y)	0.04	NA	0.9 (0.1, 10.3)	0.7 (0.1, 8.5)	0.7 (0, 11.6)	0.5 (0.1, 4)	0.2 (0, 2.2)	0.2 (0, 1.7)	0.2 (0, 1.6)	0.2 (0, 1.9)	0.2 (0, 1.3)	0.1 (0, 1.1)
Road	0.07	1.1 (0.1, 12.9)	NA	0.8 (0.1, 6.1)	0.8 (0.1, 8.9)	0.6 (0.1, 2.6)	0.3 (0.1, 1.5)	0.2 (0.1, 1.1)	0.2 (0, 1.1)	0.2 (0, 1.3)	0.2 (0, 0.8)*	0.2 (0, 0.7)*
Riparian (<1y)	0.05	1.4 (0.1, 15.9)	1.2 (0.2, 9.1)	NA	1.0 (0.1, 11.2)	0.7 (0.1, 3.1)	0.3 (0.1, 1.8)	0.3 (0.1, 1.4)	0.3 (0.1, 1.4)	0.3 (0, 1.7)	0.2 (0, 1.1)	0.2 (0, 0.9)*
Farm-stream	0.03	1.4 (0.1, 23.7)	1.3 (0.1, 14.6)	1.0 (0.1, 12.2)	NA	0.7 (0.1, 5.8)	0.3 (0, 3)	0.3 (0, 2.4)	0.3 (0, 2.3)	0.3 (0, 2.7)	0.2 (0, 1.9)	0.2 (0, 1.5)
Heath (<1y)	0.21	2 (0.2, 16.3)	1.8 (0.4, 8.2)	1.5 (0.3, 6.8)	1.4 (0.2, 11.5)	NA	0.5 (0.2, 1.5)	0.4 (0.2, 1)	0.4 (0.2, 1)	0.4 (0.1, 1.5)	0.3 (0.1, 0.8)*	0.3 (0.1, 0.7)*
Heath (>35y)	0.07	4.1 (0.5, 36.2)	3.7 (0.7, 19.4)	3 (0.5, 16.4)	2.9 (0.3, 24.6)	2 (0.7, 6.3)	NA	0.9 (0.3, 2.4)	0.8 (0.3, 2.4)	0.8 (0.2, 3.4)	0.6 (0.2, 2)	0.6 (0.2, 1.5)
Farmland	0.18	4.6 (0.6, 35.9)	4.1 (0.9, 18.5)	3.4 (0.7, 15.6)	3.2 (0.4, 24.8)	2.3 (1, 5.4)	1.1 (0.4, 3.1)	NA	0.9 (0.4, 2)	0.9 (0.2, 3.1)	0.7 (0.3, 1.7)	0.6 (0.3, 1.3)
Edge (open)	0.11	4.9 (0.6, 39.3)	4.4 (0.9, 20.6)	3.6 (0.7, 17.4)	3.4 (0.4, 27.3)	2.4 (1, 6.1)	1.2 (0.4, 3.5)	1.1 (0.5, 2.3)	NA	0.9 (0.2, 3.5)	0.8 (0.3, 1.9)	0.7 (0.3, 1.5)
Heath (7-35y)	0.03	5.4 (0.5, 55.1)	4.8 (0.8, 30)	3.9 (0.6, 25.6)	3.8 (0.4, 37.9)	2.7 (0.7, 10.4)	1.3 (0.3, 5.8)	1.2 (0.3, 4.2)	1.1 (0.3, 4.2)	NA	0.9 (0.2, 3.4)	0.8 (0.2, 2.7)
Riparian (>35y)	0.08	6.3 (0.8, 50.3)	5.6 (1.2, 26.9)*	4.6 (0.9, 22.6)	4.4 (0.5, 35.9)	3.1 (1.2, 8.1)*	1.5 (0.5, 4.7)	1.4 (0.6, 3.1)	1.3 (0.5, 3.1)	1.2 (0.3, 4.6)	NA	0.9 (0.4, 2)
Edge (forest)	0.13	7.1 (0.9, 55.5)	6.4 (1.4, 28.5)*	5.2 (1.1, 24.2)*	5 (0.7, 38.4)	3.6 (1.5, 8.2)*	1.7 (0.6, 4.7)	1.6 (0.8, 3.1)	1.5 (0.7, 3.2)	1.3 (0.4, 4.8)	1.1 (0.5, 2.5)	NA

[a] This habitat was available but never used and therefore had to be dropped from the analysis

night ($n_{observed}$ = 411)

variable	avail.	Heath (>35y)	Eucalypt (>35y)	Heath (<1y)	Eucalypt (<1y)	Road	Riparian (>35y)	Farmland	Edge (forest)	Riparian (<1y)	Farm-stream	Edge (open)	Heath (7-35y)
Heath (>35y)	0.09	NA	0.4 (0.1, 1.3)	0.3 (0.1, 0.8)*	0.3 (0.1, 0.8)*	0.2 (0.1, 0.5)*	0.2 (0.1, 0.4)*	0.2 (0.1, 0.4)*	0.2 (0.1, 0.4)*	0.2 (0.1, 0.4)*	0.1 (0.1, 0.3)*	0.1 (0.1, 0.3)*	0.1 (0, 0.2)*
Eucalypt (>35y)	0.03	2.3 (0.7, 7.1)	NA	0.8 (0.3, 1.9)	0.7 (0.2, 1.8)	0.5 (0.2, 1.1)	0.4 (0.1, 1)	0.4 (0.2, 0.9)*	0.4 (0.2, 0.9)*	0.4 (0.1, 1)*	0.3 (0.1, 0.8)*	0.3 (0.1, 0.7)*	0.2 (0.1, 0.5)*
Heath (<1y)	0.15	3 (1.3, 6.8)*	1.3 (0.5, 3.2)	NA	0.9 (0.4, 1.7)	0.6 (0.3, 1)*	0.5 (0.3, 0.9)*	0.5 (0.3, 0.7)*	0.5 (0.3, 0.7)*	0.5 (0.3, 0.9)*	0.4 (0.2, 0.7)*	0.4 (0.3, 0.6)*	0.2 (0.1, 0.4)*
Eucalypt (<1y)	0.05	3.4 (1.3, 8.9)*	1.5 (0.5, 4)	1.1 (0.6, 2.2)	NA	0.7 (0.3, 1.4)	0.6 (0.3, 1.2)	0.6 (0.3, 1)	0.5 (0.3, 1)	0.5 (0.2, 1.2)	0.5 (0.2, 0.9)*	0.5 (0.2, 0.9)*	0.3 (0.1, 0.5)*
Road	0.06	5.1 (2.2, 11.9)*	2.2 (0.9, 5.6)	1.7 (1, 2.9)*	1.5 (0.7, 3)	NA	0.9 (0.5, 1.6)	0.8 (0.5, 1.3)	0.8 (0.5, 1.3)	0.8 (0.4, 1.6)	0.7 (0.4, 1.2)	0.7 (0.4, 1.1)	0.4 (0.2, 0.7)*
Riparian (>35y)	0.04	5.9 (2.4, 14.6)*	2.6 (1, 6.7)	2 (1.1, 3.6)*	1.7 (0.8, 3.6)	1.2 (0.6, 2.2)	NA	1.0 (0.6, 1.6)	0.9 (0.5, 1.6)	0.9 (0.5, 1.9)	0.8 (0.4, 1.5)	0.8 (0.5, 1.4)	0.5 (0.2, 0.9)*
Farmland	0.24	6.2 (2.9, 13.5)*	2.7 (1.1, 6.4)*	2.1 (1.4, 3.1)*	1.8 (1, 3.4)	1.2 (0.8, 1.9)	1.0 (0.6, 1.8)	NA	1.0 (0.7, 1.4)	1.0 (0.5, 1.7)	0.8 (0.5, 1.3)	0.8 (0.6, 1.1)	0.5 (0.3, 0.8)*
Edge (forest)	0.12	6.3 (2.9, 14)*	2.8 (1.1, 6.6)*	2.1 (1.4, 3.3)*	1.9 (1, 3.5)	1.2 (0.8, 2)	1.1 (0.6, 1.9)	1.0 (0.7, 1.4)	NA	1.0 (0.5, 1.8)	0.9 (0.5, 1.4)	0.8 (0.6, 1.2)	0.5 (0.3, 0.8)*
Riparian (<1y)	0.03	6.3 (2.5, 16.1)*	2.8 (1, 7.5)*	2.1 (1.1, 4)*	1.9 (0.9, 4)	1.2 (0.6, 2.4)	1.1 (0.5, 2.2)	1.0 (0.6, 1.8)	1.0 (0.5, 1.8)	NA	0.9 (0.4, 1.7)	0.9 (0.5, 1.6)	0.5 (0.2, 1)*
Farm-stream	0.05	7.4 (3.2, 17.3)*	3.2 (1.3, 8.2)*	2.5 (1.5, 4.3)*	2.2 (1.1, 4.4)*	1.5 (0.8, 2.6)	1.3 (0.7, 2.4)	1.2 (0.8, 1.9)	1.2 (0.7, 1.9)	1.2 (0.6, 2.3)	NA	1.0 (0.6, 1.6)	0.6 (0.3, 1)
Edge (open)	0.13	7.4 (3.4, 16.4)*	3.2 (1.4, 7.8)*	2.5 (1.6, 3.8)*	2.2 (1.2, 4.1)*	1.5 (0.9, 2.4)	1.3 (0.7, 2.2)	1.2 (0.9, 1.6)	1.2 (0.8, 1.7)	1.2 (0.6, 2.1)	1.0 (0.6, 1.6)	NA	0.6 (0.3, 1)*
Heath (7-35y)	0.02	13.1 (5.4, 31.5)*	5.7 (2.2, 14.8)*	4.4 (2.5, 7.7)*	3.8 (1.8, 8)*	2.6 (1.4, 4.7)*	2.2 (1.1, 4.3)*	2.1 (1.3, 3.4)*	2.1 (1.2, 3.5)*	2.1 (1, 4.1)*	1.8 (1, 3.2)	1.8 (1.1, 2.9)*	NA

Fox 5590

day ($n_{observed}$ = 104)

variable	avail.	Farmland	Eucalypt (>35y)	Eucalypt (7-35y)	Road	Edge (open)	Edge (forest)	Riparian (7-35y)
Farmland	0.12	NA	0.1 (0, 1.7)	0.1 (0, 0.5)*	0.1 (0, 0.4)*	0 (0, 0.4)*	0 (0, 0.3)*	0 (0, 0.2)*
Eucalypt (>35y)	0.04	6.7 (0.6, 75)	NA	0.5 (0.1, 2)	0.4 (0.1, 1.7)	0.3 (0.1, 1.7)	0.3 (0.1, 1.4)	0.2 (0, 1)*
Eucalypt (7-35y)	0.53	14.5 (2, 107.6)*	2.2 (0.5, 9.4)	NA	0.8 (0.4, 1.5)	0.7 (0.2, 2)	0.6 (0.2, 1.4)	0.5 (0.3, 0.8)*
Road	0.09	18.5 (2.4, 144.3)*	2.8 (0.6, 12.9)	1.3 (0.7, 2.5)	NA	0.8 (0.3, 2.8)	0.7 (0.3, 2)	0.6 (0.3, 1.3)
Edge (open)	0.03	22 (2.4, 201)*	3.3 (0.6, 18.6)	1.5 (0.5, 4.5)	1.2 (0.4, 3.9)	NA	0.9 (0.2, 3.2)	0.7 (0.2, 2.2)
Edge (forest)	0.03	25.5 (3, 216.8)*	3.8 (0.7, 19.7)	1.8 (0.7, 4.4)	1.4 (0.5, 3.9)	1.2 (0.3, 4.4)	NA	0.8 (0.3, 2.2)
Riparian (7-35y)	0.15	30.8 (4.1, 232.5)*	4.6 (1, 20.5)*	2.1 (1.3, 3.5)*	1.7 (0.8, 3.5)	1.4 (0.5, 4.3)	1.2 (0.5, 3.1)	NA

night ($n_{observed}$ = 477)

variable	avail.	Riparian (7-35y)	Eucalypt (7-35y)	Eucalypt (>35y)	Farmland	Reservoir/dam	Edge (open)	Edge (forest)	Road
Riparian (7-35y)	0.07	NA	0.8 (0.5, 1.3)	0.6 (0.3, 1.2)	0.6 (0.3, 0.9)*	0.5 (0.2, 0.9)*	0.4 (0.2, 0.7)*	0.4 (0.2, 0.6)*	0.3 (0.2, 0.5)*
Eucalypt (7-35y)	0.27	1.3 (0.7, 2.1)	NA	0.7 (0.4, 1.3)	0.7 (0.5, 0.9)*	0.6 (0.3, 0.9)*	0.5 (0.3, 0.7)*	0.4 (0.3, 0.7)*	0.4 (0.3, 0.5)*
Eucalypt (>35y)	0.03	1.7 (0.8, 3.6)	1.4 (0.7, 2.5)	NA	1.0 (0.5, 1.7)	0.8 (0.4, 1.6)	0.6 (0.3, 1.2)	0.6 (0.3, 1.2)	0.5 (0.3, 0.9)*
Farmland	0.43	1.8 (1.1, 3)*	1.4 (1.1, 1.9)*	1.0 (0.6, 1.9)	NA	0.8 (0.5, 1.3)	0.7 (0.4, 1)*	0.6 (0.4, 0.9)*	0.5 (0.4, 0.7)*
Reservoir/dam	0.04	2.2 (1.2, 4.2)*	1.8 (1.1, 2.9)*	1.3 (0.6, 2.6)	1.2 (0.8, 2)	NA	0.8 (0.5, 1.4)	0.8 (0.5, 1.4)	0.6 (0.4, 1.1)
Edge (open)	0.05	2.7 (1.5, 5)*	2.2 (1.4, 3.4)*	1.6 (0.8, 3.1)	1.5 (1, 2.3)*	1.2 (0.7, 2.2)	NA	1.0 (0.6, 1.6)	0.8 (0.5, 1.3)
Edge (forest)	0.05	2.8 (1.6, 5)*	2.2 (1.5, 3.4)*	1.6 (0.8, 3.1)	1.6 (1.1, 2.3)*	1.3 (0.7, 2.2)	1.0 (0.6, 1.7)	NA	0.8 (0.5, 1.3)
Road	0.07	3.5 (2, 6)*	2.8 (2, 3.9)*	2 (1.1, 3.8)*	1.9 (1.4, 2.7)*	1.6 (0.9, 2.6)	1.3 (0.8, 2)	1.2 (0.8, 1.9)	NA

Fox 4590

day (n$_{observed}$ = 337)

variable	avail.	Plantation	Road	Farmland	Eucalypt (>35y)	Edge (forest)	Reservoir/dam
Plantation	0.42	NA	0.9 (0.4, 1.9)	0.8 (0.3, 2.1)	0.8 (0.6, 1.1)	0.8 (0.5, 1.3)	0.3 (0.2, 0.4)*
Road	0.03	1.1 (0.5, 2.4)	NA	0.9 (0.3, 3)	0.9 (0.4, 1.9)	0.9 (0.4, 2)	0.3 (0.1, 0.7)*
Farmland	0.02	1.2 (0.5, 3)	1.1 (0.3, 3.3)	NA	0.9 (0.4, 2.4)	0.9 (0.3, 2.5)	0.3 (0.1, 0.8)*
Eucalypt (>35y)	0.40	1.3 (0.9, 1.7)	1.1 (0.5, 2.4)	1.1 (0.4, 2.7)	NA	1.0 (0.6, 1.6)	0.3 (0.2, 0.5)*
Edge (forest)	0.05	1.3 (0.8, 2.2)	1.2 (0.5, 2.7)	1.1 (0.4, 3)	1.0 (0.6, 1.7)	NA	0.4 (0.2, 0.6)*
Reservoir/dam	0.08	3.6 (2.5, 5.4)*	3.2 (1.4, 7.2)*	3.1 (1.2, 8)*	2.9 (1.9, 4.3)*	2.8 (1.5, 5.1)*	NA

night (n$_{observed}$ = 839)

variable	avail.	Plantation	Eucalypt (>35y)	Edge (forest)	Farmland	Edge (open)	Farm-stream	Road	Reservoir
Plantation	0.38	NA	1.0 (0.7, 1.3)	0.6 (0.4, 0.9)*	0.5 (0.4, 0.6)*	0.4 (0.3, 0.7)*	0.4 (0.3, 0.5)*	0.3 (0.3, 0.4)*	0.2 (0.1, 0.2)*
Eucalypt (>35y)	0.14	1.0 (0.8, 1.4)	NA	0.6 (0.4, 1)*	0.5 (0.3, 0.6)*	0.5 (0.3, 0.7)*	0.4 (0.2, 0.6)*	0.3 (0.2, 0.4)*	0.2 (0.1, 0.3)*
Edge (forest)	0.05	1.6 (1.1, 2.3)*	1.6 (1, 2.3)*	NA	0.7 (0.5, 1)	0.7 (0.4, 1.1)	0.6 (0.4, 0.9)*	0.5 (0.4, 0.7)*	0.3 (0.2, 0.4)*
Farmland	0.22	2.2 (1.7, 2.8)*	2.2 (1.6, 3)*	1.4 (1, 2)	NA	1.0 (0.7, 1.4)	0.8 (0.6, 1.1)	0.7 (0.5, 0.9)*	0.4 (0.3, 0.5)*
Edge (open)	0.03	2.2 (1.5, 3.3)*	2.2 (1.4, 3.4)*	1.4 (0.9, 2.3)	1.0 (0.7, 1.5)	NA	0.8 (0.5, 1.3)	0.7 (0.5, 1.1)	0.4 (0.3, 0.6)*
Farm-stream	0.04	2.7 (1.9, 3.9)*	2.7 (1.8, 4.1)*	1.7 (1.1, 2.7)*	1.3 (0.9, 1.8)	1.2 (0.8, 2)	NA	0.9 (0.6, 1.3)	0.5 (0.3, 0.7)*
Road	0.1	3.1 (2.5, 4)*	3.1 (2.2, 4.2)*	2 (1.4, 2.9)*	1.4 (1.1, 1.8)*	1.4 (0.9, 2.1)	1.1 (0.8, 1.6)	NA	0.6 (0.4, 0.7)*
Reservoir	0.04	5.7 (4.3, 7.5)*	5.6 (4, 7.9)*	3.6 (2.4, 5.3)*	2.6 (2, 3.4)*	2.5 (1.7, 3.9)*	2.1 (1.4, 3.1)*	1.8 (1.3, 2.4)*	NA

Fox GAMY

day (n$_{observed}$ = 127)

variable	avail.	Road	Heath (1-7y)	Eucalypt (<1y)	Eucalypt (>35y)	Eucalypt (1-7y)	Edge (open)	Farmland	Edge (forest)	Farm-stream
Road	0.05	NA	0.9 (0.1, 10)	0.4 (0.1, 3.1)	0.4 (0.1, 1.8)	0.4 (0.1, 1.6)	0.3 (0.1, 1.3)	0.3 (0.1, 1.1)	0.2 (0.1, 1.1)	0.2 (0.1, 1)
Heath (1-7y)	0.02	1.1 (0.1, 12.6)	NA	0.5 (0, 5.5)	0.4 (0.1, 3.4)	0.4 (0.1, 3.1)	0.3 (0, 2.4)	0.3 (0, 2.3)	0.3 (0, 2.1)	0.3 (0, 2)
Eucalypt (<1y)	0.03	2.3 (0.3, 15.8)	2 (0.2, 22.6)	NA	0.8 (0.2, 3.9)	0.8 (0.2, 3.7)	0.6 (0.1, 2.8)	0.6 (0.1, 2.5)	0.6 (0.1, 2.4)	0.5 (0.1, 2.3)
Eucalypt (>35y)	0.08	2.7 (0.6, 13.1)	2.4 (0.3, 19.8)	1.2 (0.3, 5.6)	NA	1.0 (0.4, 2.5)	0.8 (0.3, 1.9)	0.7 (0.3, 1.6)	0.7 (0.3, 1.6)	0.6 (0.3, 1.5)
Eucalypt (1-7y)	0.15	2.8 (0.6, 13.1)	2.5 (0.3, 19.7)	1.3 (0.3, 5.8)	1.0 (0.4, 2.7)	NA	0.8 (0.3, 1.9)	0.8 (0.4, 1.6)	0.7 (0.3, 1.6)	0.7 (0.3, 1.6)
Edge (open)	0.11	3.6 (0.8, 16.2)	3.2 (0.4, 25)	1.6 (0.4, 6.9)	1.3 (0.5, 3.2)	1.3 (0.5, 3)	NA	1.0 (0.5, 1.8)	0.9 (0.4, 1.8)	0.8 (0.4, 1.8)
Farmland	0.36	3.7 (0.9, 15.6)	3.3 (0.4, 24.7)	1.6 (0.4, 6.7)	1.4 (0.6, 3)	1.3 (0.6, 2.8)	1.0 (0.6, 1.9)	NA	0.9 (0.5, 1.6)	0.9 (0.5, 1.6)
Edge (forest)	0.1	4.1 (0.9, 18.3)	3.7 (0.5, 28.4)	1.8 (0.4, 7.8)	1.5 (0.6, 3.6)	1.5 (0.6, 3.3)	1.1 (0.5, 2.4)	1.1 (0.6, 2)	NA	1.0 (0.5, 2)
Farm-stream	0.11	4.3 (1, 19.5)	3.8 (0.5, 30.1)	1.9 (0.4, 8.4)	1.6 (0.6, 3.9)	1.5 (0.6, 3.7)	1.2 (0.6, 2.6)	1.2 (0.6, 2.1)	1.0 (0.5, 2.2)	NA

night (n$_{observed}$ = 155)

variable	avail.	Road	Heath (<1y)	Eucalypt (<1y)	Farmland	Eucalypt (>35y)	Edge (open)	Eucalypt (1-7y)	Farm-stream	Edge (forest)
Road	0.04	NA	0.8 (0.1, 11)	0.5 (0, 6.6)	0.2 (0, 1.4)	0.2 (0, 1.3)	0.2 (0, 1.1)	0.1 (0, 0.8)*	0.1 (0, 0.7)*	0.1 (0, 0.7)*
Heath (<1y)	0.04	1.2 (0.1, 15.4)	NA	0.6 (0.1, 4.2)	0.2 (0, 1.3)	0.2 (0, 1.2)	0.2 (0, 1)	0.1 (0, 0.7)*	0.1 (0, 0.6)*	0.1 (0, 0.6)*
Eucalypt (<1y)	0.02	2 (0.2, 26.5)	1.7 (0.2, 12.2)	NA	0.4 (0.1, 2.2)	0.3 (0.1, 2)	0.3 (0.1, 1.8)	0.2 (0, 1.3)	0.2 (0, 1.1)	0.2 (0, 1)
Farmland	0.34	5.2 (0.7, 38.2)	4.4 (0.8, 24.1)	2.6 (0.5, 14.4)	NA	0.9 (0.4, 1.8)	0.8 (0.4, 1.4)	0.6 (0.3, 1.1)	0.5 (0.3, 0.8)*	0.5 (0.3, 0.8)*
Eucalypt (>35y)	0.07	6 (0.8, 47.8)	5.1 (0.9, 30.3)	3 (0.5, 18.1)	1.2 (0.6, 2.4)	NA	0.9 (0.4, 2)	0.6 (0.3, 1.6)	0.6 (0.3, 1.2)	0.6 (0.3, 1.2)
Edge (open)	0.12	6.6 (0.9, 50.2)	5.6 (1, 32)	3.3 (0.6, 19.1)	1.3 (0.7, 2.2)	1.1 (0.5, 2.4)	NA	0.7 (0.3, 1.6)	0.6 (0.3, 1.1)	0.6 (0.3, 1.1)
Eucalypt (1-7y)	0.17	9.3 (1.2, 71.8)*	7.9 (1.3, 46.1)*	4.6 (0.8, 28.1)	1.8 (0.9, 3.7)	1.5 (0.6, 3.7)	1.4 (0.6, 3.1)	NA	0.9 (0.4, 1.9)	0.9 (0.4, 1.8)
Farm-stream	0.11	10.8 (1.4, 81)*	9.1 (1.6, 51.5)*	5.4 (0.9, 30.8)	2.1 (1.2, 3.5)*	1.8 (0.8, 3.9)	1.6 (0.9, 3)	1.2 (0.5, 2.5)	NA	1.0 (0.5, 1.8)
Edge (forest)	0.09	10.9 (1.4, 82)*	9.2 (1.7, 51.5)*	5.4 (1, 30.8)	2.1 (1.2, 3.6)*	1.8 (0.8, 3.9)	1.6 (0.9, 3.1)	1.2 (0.5, 2.5)	1.0 (0.6, 1.9)	NA

Fox DOUG

day ($n_{observed}$ = 187)

variable	avail.	Farmland	Reservoir/dam	Eucalypt (7-35y)	Road	Edge (forest)	Riparian (<1y)	Heath (7-35y)	Eucalypt (<1y)
Riparian (7-35y) [a]	0.02								
Farmland	0.09	NA	0.9 (0.1, 8.5)	0.3 (0.1, 1.1)	0.2 (0.1, 0.7)*	0.2 (0.1, 0.7)*	0.2 (0, 0.6)*	0.2 (0, 0.6)*	0.1 (0, 0.2)*
Reservoir/dam	0.02	1.2 (0.1, 11.3)	NA	0.4 (0, 2.9)	0.2 (0, 1.8)	0.2 (0, 1.8)	0.2 (0, 1.5)	0.2 (0, 1.5)	0.1 (0, 0.6)*
Eucalypt(7-35y)	0.19	3.1 (0.9, 10.9)	2.7 (0.3, 20.9)	NA	0.6 (0.3, 1.2)	0.6 (0.3, 1.4)	0.5 (0.2, 1.1)	0.5 (0.2, 1.2)	0.2 (0.1, 0.4)*
Road	0.12	4.9 (1.4, 17.4)*	4.3 (0.6, 33.3)	1.6 (0.8, 3.1)	NA	1.0 (0.4, 2.3)	0.8 (0.4, 1.8)	0.7 (0.3, 1.9)	0.4 (0.2, 0.6)*
Edge (forest)	0.05	5.2 (1.4, 19.9)*	4.5 (0.6, 37)	1.7 (0.7, 4)	1.1 (0.4, 2.5)	NA	0.9 (0.3, 2.2)	0.8 (0.3, 2.3)	0.4 (0.2, 0.8)*
Riparian (<1y)	0.08	6 (1.6, 22.1)*	5.2 (0.6, 41.4)	1.9 (0.9, 4.2)	1.2 (0.6, 2.6)	1.1 (0.4, 2.9)	NA	0.9 (0.3, 2.5)	0.4 (0.2, 0.8)*
Heath (7-35y)	0.05	6.6 (1.6, 27.8)*	5.8 (0.7, 49.9)	2.1 (0.9, 5.4)	1.3 (0.5, 3.4)	1.3 (0.4, 3.8)	1.1 (0.4, 3.1)	NA	0.5 (0.2, 1.2)
Eucalypt (<1y)	0.4	13.8 (4.2, 45.3)*	12 (1.6, 89.2)*	4.4 (2.6, 7.7)*	2.8 (1.6, 4.8)*	2.7 (1.2, 5.7)*	2.3 (1.2, 4.3)*	2.1 (0.9, 5)	NA

night ($n_{observed}$ = 296)

variable	avail.	Eucalypt (7-35y)	Farm-stream	Riparian (<1y)	Farmland	Eucalypt (<1y)	Road	Edge (forest)	Reservoir/dam	Edge (open)
Riparian (7-35y) [a]	0.03									
Eucalypt (7-35y)	0.17	NA	1.0 (0.3, 3.3)	0.8 (0.4, 1.8)	0.5 (0.3, 0.8)*	0.4 (0.2, 0.7)*	0.3 (0.2, 0.6)*	0.3 (0.1, 0.5)*	0.3 (0.1, 0.5)*	0.2 (0.1, 0.4)*
Farm-stream	0.02	1.0 (0.3, 3.6)	NA	0.9 (0.2, 3.3)	0.5 (0.2, 1.6)	0.4 (0.1, 1.4)	0.4 (0.1, 1.2)	0.3 (0.1, 1)*	0.3 (0.1, 0.9)*	0.2 (0.1, 0.7)*
Riparian (<1y)	0.06	1.2 (0.6, 2.6)	1.2 (0.3, 4.3)	NA	0.6 (0.3, 1.2)	0.5 (0.2, 0.9)*	0.4 (0.2, 0.9)*	0.3 (0.1, 0.7)*	0.3 (0.1, 0.7)*	0.3 (0.1, 0.5)*
Farmland	0.25	2.1 (1.3, 3.6)*	2 (0.6, 6.6)	1.8 (0.9, 3.6)	NA	0.8 (0.6, 1.3)	0.7 (0.5, 1.2)	0.6 (0.3, 0.9)*	0.5 (0.3, 0.9)*	0.4 (0.3, 0.7)*
Eucalypt (<1y)	0.24	2.5 (1.5, 4.2)*	2.4 (0.7, 8)	2.1 (1.1, 4.1)*	1.2 (0.8, 1.8)	NA	0.9 (0.6, 1.3)	0.7 (0.4, 1.1)	0.6 (0.4, 1.1)	0.5 (0.3, 0.9)*
Road	0.1	2.9 (1.7, 5)*	2.8 (0.8, 9.3)	2.4 (1.2, 5)*	1.4 (0.9, 2.2)	1.1 (0.7, 1.8)	NA	0.8 (0.5, 1.4)	0.7 (0.4, 1.3)	0.6 (0.4, 1)
Edge (forest)	0.05	3.7 (2.1, 6.8)*	3.6 (1.1, 12)*	3.1 (1.4, 6.7)*	1.7 (1.1, 2.9)*	1.5 (0.9, 2.4)	1.3 (0.7, 2.2)	NA	0.9 (0.5, 1.8)	0.8 (0.5, 1.4)
Reservoir/dam	0.04	4 (2.1, 7.8)*	3.8 (1.1, 13.2)*	3.3 (1.5, 7.5)*	1.9 (1.1, 3.2)*	1.6 (0.9, 2.8)	1.4 (0.7, 2.5)	1.1 (0.6, 2)	NA	0.8 (0.5, 1.5)
Edge (open)	0.06	4.7 (2.7, 8.5)*	4.5 (1.4, 15.1)*	3.9 (1.8, 8.4)*	2.2 (1.4, 3.5)*	1.9 (1.2, 3)*	1.6 (1, 2.7)	1.3 (0.7, 2.2)	1.2 (0.6, 2.2)	NA

[a] This habitat was available but never used and therefore had to be dropped from the analysis

Fox 4400

day ($n_{observed}$ = 240)

variable	avail.	Reservoir/dam	Farmland	Road	Plantation	Farm-stream	Eucalypt (7-35y)	Eucalypt (>35y)	Edge (open)	Edge (forest)
Reservoir/dam	0.04	NA	0.9 (0.3, 2.3)	0.7 (0.2, 2.1)	0.6 (0.2, 2.2)	0.5 (0.2, 1.9)	0.3 (0.1, 0.9)*	0.3 (0.1, 0.8)*	0.2 (0.1, 0.7)*	0.2 (0.1, 0.7)*
Farmland	0.35	1.2 (0.4, 3.2)	NA	0.8 (0.4, 1.5)	0.7 (0.3, 1.8)	0.6 (0.3, 1.4)	0.3 (0.2, 0.7)*	0.3 (0.2, 0.6)*	0.3 (0.2, 0.5)*	0.3 (0.2, 0.4)*
Road	0.06	1.5 (0.5, 4.8)	1.3 (0.7, 2.6)	NA	0.9 (0.3, 2.6)	0.8 (0.3, 2.2)	0.4 (0.2, 1)	0.4 (0.2, 0.9)*	0.4 (0.2, 0.8)*	0.4 (0.2, 0.7)*
Plantation	0.05	1.6 (0.5, 5.8)	1.4 (0.6, 3.4)	1.1 (0.4, 3)	NA	0.9 (0.3, 2.8)	0.5 (0.2, 1.2)	0.5 (0.2, 1)	0.4 (0.1, 1)*	0.4 (0.2, 0.9)*
Farm-stream	0.03	1.9 (0.5, 6.7)	1.6 (0.7, 3.7)	1.2 (0.4, 3.4)	1.2 (0.4, 3.7)	NA	0.5 (0.2, 1.5)	0.5 (0.2, 1.4)	0.4 (0.2, 1.1)	0.4 (0.2, 1.1)
Eucalypt (7-35y)	0.1	3.5 (1.1, 11.3)*	3 (1.4, 6.4)*	2.3 (1, 5.7)	2.2 (0.9, 5.6)	1.9 (0.7, 5.5)	NA	1.0 (0.6, 1.7)	0.8 (0.4, 1.9)	0.8 (0.4, 1.8)
Eucalypt (>35y)	0.27	3.6 (1.2, 10.2)*	3 (1.7, 5.3)*	2.3 (1.1, 5)*	2.2 (1, 5)	1.9 (0.7, 4.9)	1.0 (0.6, 1.7)	NA	0.8 (0.4, 1.6)	0.8 (0.5, 1.5)
Edge (open)	0.05	4.3 (1.5, 12.6)*	3.7 (2.2, 6.2)*	2.8 (1.3, 6.1)*	2.6 (1, 6.8)*	2.3 (0.9, 5.8)	1.2 (0.5, 2.7)	1.2 (0.6, 2.3)	NA	1.0 (0.6, 1.8)
Edge (forest)	0.06	4.3 (1.5, 12.4)*	3.7 (2.3, 6.1)*	2.8 (1.4, 6)*	2.7 (1.1, 6.6)*	2.3 (0.9, 5.7)	1.2 (0.6, 2.6)	1.2 (0.7, 2.2)	1.0 (0.6, 1.8)	NA

night ($n_{observed}$ = 765)

variable	avail.	Plantation	Reservoir/dam	Eucalypt (7-35y)	Eucalypt (>35y)	Road	Edge (open)	Edge (forest)	Farmland	Farm-stream
Plantation	0.04	NA	0.4 (0.2, 1)	0.4 (0.2, 0.9)*	0.4 (0.2, 0.8)*	0.3 (0.2, 0.6)*	0.3 (0.1, 0.6)*	0.3 (0.1, 0.6)*	0.3 (0.1, 0.5)*	0.2 (0.1, 0.4)*
Reservoir/dam	0.02	2.2 (1, 5.2)	NA	1.0 (0.5, 2.1)	0.9 (0.5, 1.6)	0.7 (0.4, 1.3)	0.7 (0.3, 1.3)	0.6 (0.3, 1.2)	0.6 (0.3, 1.1)	0.4 (0.2, 0.8)*
Eucalypt (7-35y)	0.04	2.3 (1.1, 4.9)*	1.0 (0.5, 2.2)	NA	0.9 (0.5, 1.5)	0.7 (0.4, 1.2)	0.7 (0.4, 1.3)	0.7 (0.4, 1.2)	0.6 (0.4, 1.1)	0.4 (0.2, 0.8)*
Eucalypt (>35y)	0.13	2.5 (1.3, 4.9)*	1.1 (0.6, 2.1)	1.1 (0.7, 1.8)	NA	0.8 (0.5, 1.2)	0.8 (0.5, 1.2)	0.7 (0.5, 1.1)	0.7 (0.5, 1)*	0.5 (0.3, 0.8)*
Road	0.06	3.3 (1.7, 6.6)*	1.5 (0.8, 2.8)	1.4 (0.8, 2.6)	1.3 (0.9, 2)	NA	1.0 (0.6, 1.5)	0.9 (0.6, 1.5)	0.9 (0.6, 1.2)	0.6 (0.4, 1)*
Edge (open)	0.07	3.4 (1.7, 6.7)*	1.5 (0.8, 2.9)	1.5 (0.8, 2.7)	1.3 (0.9, 2.1)	1.0 (0.7, 1.5)	NA	1.0 (0.6, 1.5)	0.9 (0.7, 1.2)	0.6 (0.4, 1)*
Edge (forest)	0.06	3.5 (1.7, 7)*	1.6 (0.8, 3)	1.5 (0.8, 2.8)	1.4 (0.9, 2.1)	1.1 (0.7, 1.6)	1.0 (0.7, 1.6)	NA	0.9 (0.7, 1.3)	0.7 (0.4, 1)
Farmland	0.54	3.7 (2, 7.1)*	1.7 (0.9, 3)	1.6 (0.9, 2.8)	1.5 (1, 2.1)*	1.1 (0.8, 1.6)	1.1 (0.8, 1.5)	1.1 (0.8, 1.5)	NA	0.7 (0.5, 1)*
Farm-stream	0.04	5.3 (2.6, 10.8)*	2.4 (1.2, 4.6)*	2.3 (1.2, 4.4)*	2.1 (1.3, 3.4)*	1.6 (1, 2.5)*	1.6 (1, 2.4)*	1.5 (1, 2.4)	1.4 (1, 2)*	NA

Supplementary Table S5. Procedure and permit details for red fox *Vulpes vulpes* research in the Otway and Annya regions, Victoria, Australia.

Item	Otway	Annya
Trap-check frequency	12 hour (traps were checked more frequently in hot or inclement weather)	24 hour (traps were checked more frequently in hot or inclement weather)
Sedation	0.05 mg/kg medetomidine (Domitor, Zoetis Australia) and 3mg/kg teletamine-zolazepam (Zoletil 100, Zoetis Australia)	0.05 mg/kg medetomidine (Domitor, Zoetis Australia)
Sedation reversal agent	0.025 mg/kg atipamezole (Antisedan, Zoetis Australia)	0.025 mg/kg atipamezole (Antisedan, Zoetis Australia)
GPS collar specifications	*2.7 – 4.2 kg foxes*: 134 g GPS collar (Telemetry Solutions *Quantum 4000E Small Collar* with remote download, drop-off mechanism and activity sensor, www.telemetrysolutions.com)<br>*≥ 4.3 kg foxes*: 215 g GPS collar (FollowIt AB *Tellus Ultra Light GPS collar* with remote UHF & GSM download, drop-off mechanism and activity sensor, www.followit.se)	*> 4.0 kg foxes*: 120 g GPS collar with drop-off mechanism (Sirtrack, www.sirtrack.com)
GPS fix schedule	*Session one*: rotating variable schedule (to produce 36 fixes over 12 days at 2.6 hour intervals), interspersed by 24 hour periods of intense data collection at 15 min intervals.<br>Sessions two and three: 30 min intervals	*Sessions four and five*:<br>60 min intervals (from mid-January 2015)
Data retrieval	*Telemetry Solutions collars:* via UHF remote download and/or direct download after collar recovery.<br>*Followit collars:* via UHF remote download, GSM mobile signal and/or direct download after collar recovery	*Sirtrack collars:* direct download after collar recovery
GPS collar drop-off	*Telemetry Solutions collars*: scheduled in advance based on predicted collar battery life<br>*Followit collars*: triggered remotely via UHF when low-battery signal received	*Sirtrack collars:* scheduled in advance for 31 June 2015
Data screening. Fixes were removed if, from a window of 10 fixes, they were:	*Session one (15 min intervals):* >2500 m from median or >1500 m from mean<br>*Sessions two and three (30 min intervals):* >5000 m from median or > 3000 m from mean	*Sessions four and five (60 min intervals):* >10000 m from median or > 6000 m from mean
Animal Ethics approval	University of Melbourne Animal Ethics Committee (AEC 1011632 and AEC 132854)	Department of Environment, Land, Water and Planning Animal Ethics Committee (AEC 11/22)
Research permits (Victorian Government Department of Environment and Primary Industries)	Research permits 10005514 and 10006882	Research permit 10007449
Pest animal permit	RE71	RE84

Supplementary Table S6. Habitat types in study landscape.

The landscape was categorised into 20 distinct habitat types according to a classification hierarchy, with land that was not identified as the first habitat type being identified as one of the subsequent habitat types. All mapping data were obtained from www.data.vic.gov.au unless otherwise specified. *Derivation* specifies the map layer name and subsequent modifications using ArcMap 10.2.1 (ESRI 2014)

Habitat type	Description	Derivation
Urban	Land within the 'urban-residential' layer or within 20 m of a house or building outside this area	LANDUSE100 (clipped to urban-residential) (+ 20 m buffer)<br>VMFEAT_BUILDING_POINT (+ 20 m buffer)
Road	Land within 20 m of a road	TR_ROAD (+ 20 m buffer)
Reservoir/dam/ waterbody	Land identified as a waterbody or within 20 m of a waterbody	HY_WATER_AREA_POLYGON (+ 20 m buffer)
Edge (open)	Land without tree cover but within 20 m of an area with tree cover	TREE_DENSITY – scattered<br>TREE_DENSITY – medium<br>TREE_DENSITY – dense<br>LANDUSE_2014 subset to LU_Desc = Softwood Plantation, Hardwood Plantation, or Forestry (Commercial Timber Production)<br>We aggregated these four layers to obtain a 'tree cover' layer. We then created a 20m-wide buffer along the outside of the tree cover layer to identify 'edge (open)'
Edge (forest)	Land with tree cover (plantation or native) but within 20 m of an area without tree/plantation cover	Using the aggregated tree cover layer, we created a 20 m wide buffer along the inside to identify 'edge (forest)'
Plantation	Land with commercial plantation (softwood or hardwood) that was further than 20 m from an open edge.	LANDUSE_2014 subset to LU_Desc = Softwood Plantation, Hardwood Plantation, or Forestry (Commercial Timber Production)

[continued next page]

Forest (12 types):<br>• Recently burnt heathy woodland<br>• Young heathy woodland<br>• Mid-age heathy woodland<br>• Long-unburnt heathy woodland<br>• Recently-burnt eucalypt forest<br>• Young eucalypt forest<br>• Mid-age eucalypt forest<br>• Long-unburnt eucalypt forest<br>• Recently-burnt riparian forest<br>• Young riparian forest<br>• Mid-age riparian forest<br>• Long-unburnt riparian forest	Land within the tree cover layer that was further than 20 m from an open edge. Forest was grouped into three vegetation types according to ecological vegetation class (EVC) and landscape position.<br>The time since fire (adjusted for data collection date) was also determined.	Native Vegetation - Modelled 2005 Ecological Vegetation Classes (with Bioregional Conservation Status)<br>Time since fire [provided by the Department of Environment, Land, Water and Planning]<br>We clipped these layers so that their extent matched that previously identified as having non-plantation tree cover that was > 20 m from the tree edge. We then grouped EVCs into three broad categories:<br>• eucalypt forest/woodland<br>• heathy woodland/heath<br>• riparian forest/woodland<br>And time since fire into four categories:<br>• recently burnt (< 1 year post-fire)<br>• young (1 - 7 years post-fire)<br>• mid-age (7 - 35 years post-fire)<br>• long-unburnt (> 35 years post-fire).<br>These layers were specific to each fox so that age classes were appropriate for the tracking period.
Farm stream	Land not identified as treed and within 20 m of a stream	HY_WATERCOURSE (+ 20 m buffer)
Farmland	Land not identified as treed and not falling within any of the previous categories (visually checked to confirm the vast majority was farmland)	

Supplementary Table S7. R packages and citations for GPS data analysis.

Package	Citation
General data manipulation	
plyr	Wickham H. (2011). The split-apply-combine strategy for data analysis. *Journal of Statistical Software*, 40(1), 1-29. URL: http://www.jstatsoft.org/v40/i01/.
raster	Hijmans RJ. (2015). raster: Geographic Data Analysis and Modeling. R package version 2.5-2. https://CRAN.R-project.org/package=raster
rgdal	Bivand R, Keitt T, Rowlingson B. (2015). rgdal: Bindings for the Geospatial Data Abstraction Library. R package version 1.1-3. https://CRAN.R-project.org/package=rgdal
rgeos	Bivand R, Rundel C. (2015). rgeos: Interface to Geometry Engine - Open Source (GEOS). R package version 0.3-15. https://CRAN.R-project.org/package=rgeos
Broad-scale resource availability	
adehabitatHR	Calenge C. (2006) The package adehabitat for the R software: a tool for the analysis of space and habitat use by animals. Ecological Modelling, 197, 516-519
ggbiplot	Vu VQ. (2011). ggbiplot: A ggplot2 based biplot. R package version 0.55. http://github.com/vqv/ggbiplot
SpatialTools	French J. (2015). SpatialTools: Tools for Spatial Data Analysis. R package version 1.0.2. https://CRAN.R-project.org/package=SpatialTools
Broad-scale habitat selection	
BBMM	Nielson RM, Sawyer H, McDonald T.L. (2013) BBMM: Browian bridge movement model. R package version 3.0
boot	Canty A, Ripley B (2015). boot: Bootstrap R (S-Plus) Functions. R package version 1.3-17. Davison AC, Hinkley DV. (1997) Bootstrap Methods and Their Applications. Cambridge University Press, Cambridge. ISBN 0-521-57391-2
adehabitatHR & adehabitatLT	Calenge C. (2006) The package adehabitat for the R software: a tool for the analysis of space and habitat use by animals. Ecological Modelling, 197, 516-519
maptools	Bivand R, Lewin-Koh N. (2015). maptools: Tools for Reading and Handling Spatial Objects. R package version 0.8-37. https://CRAN.R-project.org/package=maptools
SpatialTools	French J. (2015). SpatialTools: Tools for Spatial Data Analysis. R package version 1.0.2. https://CRAN.R-project.org/package=SpatialTools
Fine-scale habitat selection	
adehabitatLT	Calenge C. (2006) The package adehabitat for the R software: a tool for the analysis of space and habitat use by animals. Ecological Modelling, 197, 516-519
Hmisc	Harrell FE with contributions from Dupont C and many others. (2015). Hmisc: Harrell Miscellaneous. R package version 3.17-1. https://CRAN.R-project.org/package=Hmisc
maptools	Bivand R, Lewin-Koh N. (2015). maptools: Tools for Reading and Handling Spatial Objects. R package version 0.8-37. https://CRAN.R-project.org/package=maptools
sp	Pebesma, EJ, Bivand RS. (2005). Classes and methods for spatial data in R. R News 5 (2), http://cran.r-project.org/doc/Rnews/

support.CEs	Aizaki H (2012). Basic functions for supporting an implementation of choice experiments in R. Journal of Statistical Software, Code Snippets, 50(2), 1-24. URL http://www.jstatsoft.org/v50/c02/
survival	Therneau T (2015). A Package for Survival Analysis in S version 2.38, <http://CRAN.R-project.org/package=survival>.
	Therneau TM, Grambsch PM (2000)._Modeling Survival Data: Extending the Cox Model_.Springer, New York. ISBN 0-387-98784-3.
